# A phase I clinical trial of oncolytic adenovirus mediated suicide and interleukin-12 gene therapy in patients with recurrent localized prostate adenocarcinoma

**DOI:** 10.1371/journal.pone.0291315

**Published:** 2023-09-15

**Authors:** Shyam Nyati, Hans Stricker, Kenneth N. Barton, Pin Li, Mohamed Elshaikh, Haythem Ali, Stephen L. Brown, Clara Hwang, James Peabody, Svend O. Freytag, Benjamin Movsas, Farzan Siddiqui

**Affiliations:** 1 Department of Radiation Oncology, Henry Ford Cancer Institute, Henry Ford Health, Detroit, Michigan, United States of America; 2 Department of Radiology, College of Osteopathic Medicine, Michigan State University, East Lansing, Michigan, United States of America; 3 Vattikuti Urology Institute, Henry Ford Cancer Institute, Henry Ford Health, Detroit, Michigan, United States of America; 4 Department of Public Health Sciences, Henry Ford Cancer Institute, Henry Ford Health, Detroit, Michigan, United States of America; 5 Department of Internal Medicine, Henry Ford Cancer Institute, Henry Ford Health, Detroit, Michigan, United States of America; 6 College of Human Medicine, Michigan State University, East Lansing, Michigan, United States of America; The University of Burdwan, INDIA

## Abstract

In a phase I dose escalation and safety study (NCT02555397), a replication-competent oncolytic adenovirus expressing yCD, TK and hIL-12 (Ad5-yCD/*mut*TK_SR39_*rep*-hIL-12) was administered in 15 subjects with localized recurrent prostate cancer (T1c-T2) at increasing doses (1 × 10^10^, to 1 × 10^12^ viral particles) followed by 7-day treatment of 5-fluorocytosine (5-FC) and valganciclovir (vGCV). The primary endpoint was toxicity through day 30 while the secondary and exploratory endpoints were quantitation of IL-12, IFNγ, CXCL10 and peripheral blood mononuclear cells (PBMC). The study maximum tolerated dose (MTD) was not reached indicating 10^12^ viral particles was safe. Total 115 adverse events were observed, most of which (92%) were grade 1/2 that did not require any treatment. Adenoviral DNA was detected only in two patients. Increase in IL-12, IFNγ, and CXCL10 was observed in 57%, 93%, and 79% patients, respectively. Serum cytokines demonstrated viral dose dependency, especially apparent in the highest-dose cohorts. PBMC analysis revealed immune system activation after gene therapy in cohort 5. The PSA doubling time (PSADT) pre and post treatment has a median of 1.55 years vs 1.18 years. This trial confirmed that replication-competent Ad5-IL-12 adenovirus (Ad5-yCD/*mut*TK_SR39_*rep*-hIL-12) was well tolerated when administered locally to prostate tumors.

## Introduction

Approximately 268,000 men are expected to be diagnosed with prostate cancer in USA in 2022 resulting in 34,500 deaths [1]. Nearly one-third of patients will elect radiation therapy (external beam and/or brachytherapy) as their primary treatment [2]. Radiotherapy provides exceptional long-term survival and disease control for men with localized, low-risk disease (Stage T1/T2, Gleason ≤ 6, PSA < 10 ng/mL) [2]. However, local recurrence rates are a challenge especially with more aggressive forms of the disease with higher Gleason scores and PSA (Stage ≥ T3, Gleason ≥ 7, PSA > 10 ng/mL). Fortunately, the rate of occult distal failure following definitive radiotherapy is < 10% at 10 years [3]. Hence, there is a substantial window of curative opportunity in men with localized recurrent disease.

Men with locally recurrent prostate cancer after definitive radiotherapy have few therapeutic options that can potentially eliminate the tumor with acceptable safety. Other than expectant management and systemic therapy (i.e., androgen suppression therapy, AST), there are few local therapeutic options for locally recurrent prostate cancer such as salvage radical prostatectomy, salvage cryoablation, salvage brachytherapy, and salvage high-intensity focused ultrasound. Although some have demonstrated encouraging 5-year disease-free survival rates in single institution studies, most are associated with some morbidity and there are no prospective randomized studies demonstrating their long-term effectiveness.

Several oncolytic adenovirus-based cytotoxic gene therapy vectors are currently being evaluated in clinical trials. Delivery of a conditionally cytotoxic gene, termed a “suicide gene” to the tumor is usually accomplished by direct intratumoral or systemic injection of a viral vector containing the suicide gene. Two suicide genes that have been evaluated in preclinical models and in the clinic are cytosine deaminase (CD) from yeast or *E*. *coli* and HSV thymidine kinase (TK), which convert non-toxic pro-drugs (5-FC and GCV, respectively) into potent agents that interfere with normal DNA synthesis [3,4]. The toxicity and efficacy of oncolytic adenovirus-mediated cytotoxic gene therapy has been evaluated in five clinical trials in prostate cancer, including a prospective randomized phase-II study by our group [5–14].

Phagocytes and dendritic cells secrete interleukin-12 (IL-12) in response to pathogens. Subsequently, IL-12 activates innate (natural killer, NK, and natural killer T lymphocytes, NKT) and adaptive (CD4+ Th and CD8+ Tc cells) immune system, causes IFN-γ secretion, enhances antigen presentation, and inhibits tumor angiogenesis.

IL-12 has demonstrated significant anti-tumor activity in preclinical models including in prostate cancer [15]. Systemic administration of IL-12 was toxic in clinical trials [16,17] while it provided modest clinical benefits when administered as a recombinant protein or expressed from retrovirally-transduced fibroblasts [18]. In a recently published clinical trial in metastatic pancreatic cancer patients, IL-12 delivered through replication-competent adenovirus (Ad5-IL-12) was found to be safe, eliciting an immune response [19] while potentially providing survival benefits [20]. In a preclinical model of prostate cancer, we demonstrated that inclusion of IL-12 to our adenoviral vector provided better tumor control at local and distant sites which was mediated by both the innate and adaptive arms of immunity [7]. We hypothesized that local administration of IL-12 expressing adenovirus should result in a high, local IL-12 concentration (in the prostate gland) leading to an improvement in anti-tumor activity without increasing toxicity. Mechanistically, the strategy of using a multi-modality approach (oncolytic viral therapy, cytotoxic gene therapy, IL-12 gene therapy), was reasoned to have the potential to cause substantial cancer cell eradication and secretion of tumor antigens. Increased tumor antigen was expected to facilitate antigen cross-presentation by professional APCs, which, when coupled with IL-12’s ability to promote Th1 differentiation, was expected to lead to the development of antigen-specific, cell-mediated immunity.

Consequently, we conducted an investigator-initiated phase I dose-escalation clinical trial to evaluate the maximum tolerated dose and safety of Ad5-IL-12 in men with recurrent localized prostate cancer. These men were administered Ad5-IL-12 directly into the prostate tumor at one of five escalating doses. Ad5-IL-12 viral replication, shedding and persistence was measured in patient blood (over time) by PCR. We also assessed the effect of the gene therapy on innate and adaptive immune system by quantitating peripheral blood mononuclear cells (PBMCs) before and after treatment with Ad5-IL-12 by flow cytometry. Additionally, IL-12, INFγ, and CXCL10 levels were measured in patient serum. Finally, the efficacy of the Ad5-IL-12 cytotoxic gene therapy in patients with recurrent prostate cancer was accessed by measuring patient survival.

## Results

### Study design and patient baseline characteristics

**Fig 1:**
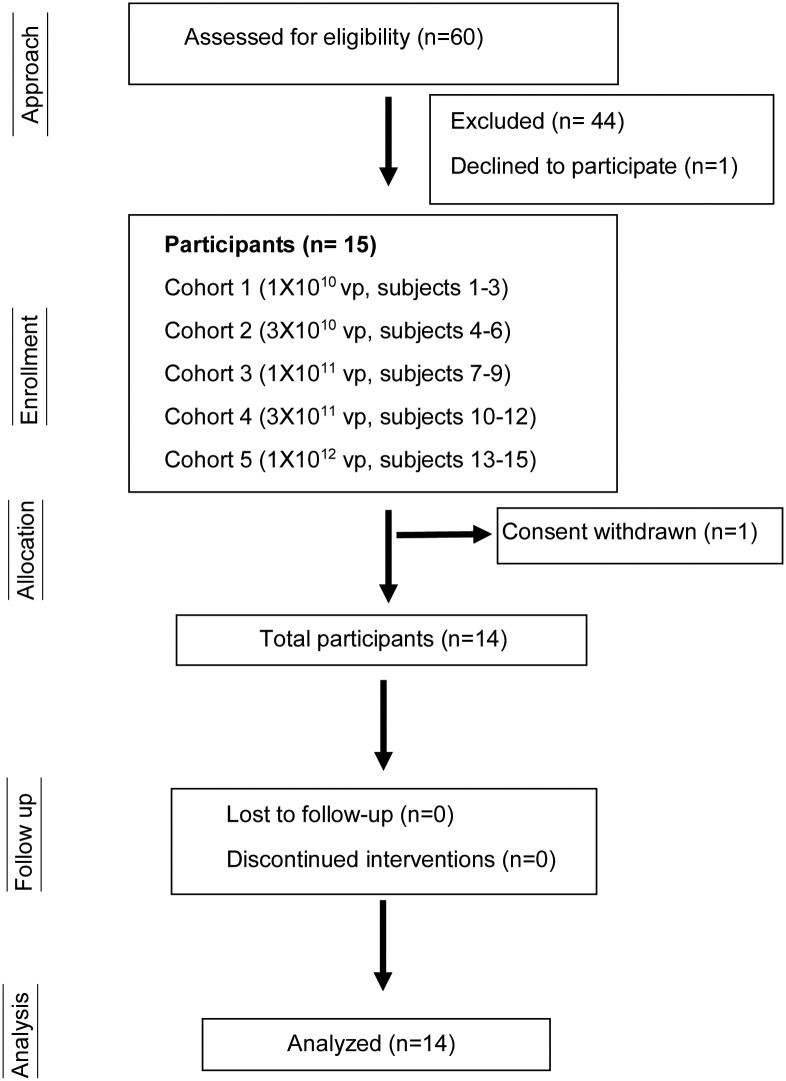
CONSORT flow diagram. Total of 60 subjects were accessed for eligibility, out of which 44 were excluded and 1 declined to participate. Remaining 15 subjects participated in this phase-1 clinical study. The subjects were recruited in five different cohorts (n = 3) who were administered with increasing doses of the Ad5-yCD/*mut*TK_SR39_*rep*-hIL-12 adenovirus. One patient withdrew consent, the data analyzed and presented here represents 14 patients.

**Fig 2:**
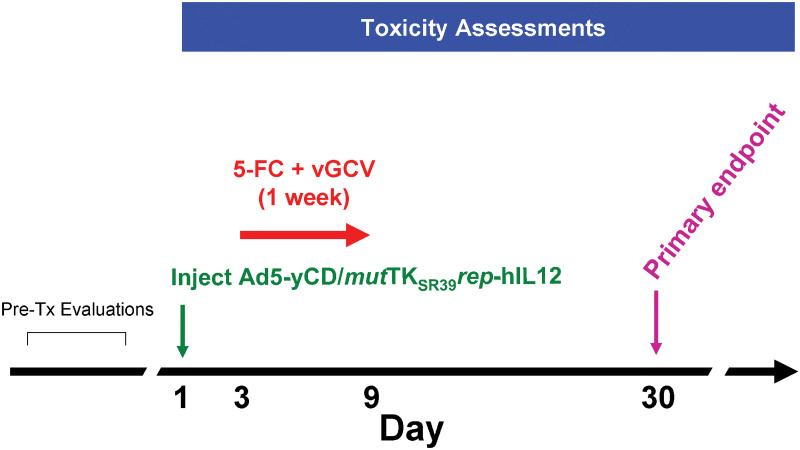
Treatment schema. Subjects underwent a series of pretreatment evaluations and met all the eligibility criteria before enrolled in the study. Subjects received a single intraprostatic injection of the Ad5-yCD/*mut*TK_SR39_*rep*-hIL-12 adenovirus at one of five dose levels (see Table 1) on day 1. Two days later (day 3), subjects received a one-week (7 day) course of 5-FC (150 mg/kg/day) + vGCV (1,800 mg/day) prodrug therapy. Toxicity assessments occurred twice a week during the first two weeks and then at every scheduled follow-up visit. The primary endpoint was toxicity through day 30.

All patients had recurrent prostate cancer following at least one cycle of radiation therapy. Patients received an intraprostatic injection of the Ad5-IL-12 adenovirus on day 1 at doses ranging from 1 × 10^10^ vp to 1 × 10^12^ vp (NCT02555397; Table 1 and Figs 1 and 2). Beginning on day 3 (day 1 being the day of Ad5-IL-12 adenoviral administration) patients started 5-FC and vGCV prodrugs for 1 week (7 days). A total of 15 patients in 5 cohorts were treated (Table 1). One patient (Patient #3) withdrew from the study on day 3. The median follow-up was 31 months (range 15–56 months). The median follow up was calculated from the date of adenoviral injection. The mean Gleason score was 7 while the most patients represented with T1c clinical stage. The mean PSA at the beginning of the gene therapy (Pre-Tx PSA) was 9.2 ng/ml (range 2.8–22.3 ng/ml). The adenovirus was deposited in all six sextants in all the patients except one patient in cohort 4 (Patient #12, 3 x 10^11^vp). The adenovirus dose distribution was skewed toward sextants with higher-grade (Gleason ≥7) cancer.

**Table 1:** Patient baseline characteristics and adenoviral injection. Each cohort had 3 patients that were injected with an increasing Ad5-IL-12 dose.

Cohort and AdV dose	Patient	Age (Yr)	Race^a^	Stage^b^	Gleason	Pre-T PSA^c^ (ng/mL)
**1**, 1X10^10^vp	1	77	W	T1c	8 (4+4)	4
	2	75	W	T1c	10 (5+5)	2.8
	3	85	W	T1c	8 (4+4)	22.3
**2**, 3X10^10^vp	4	68	AA	T1c	7 (4+3)	3.3
	5	68	AA	T2	7	3.8
	6	77	W	T1c	9 (4+5)	11.5
**3**, 1X10^11^vp	7	74	AsA	T1c	8 (4+4)	3.6
	8	66	AA	T2	8 (4+4)	6.6
	9	83	AA	T1c	7 (3+4)	10.4
**4**, 3X10^11^vp	10	70	AA	T2		3.1
	11	75	AA	T1c	9 (4+5)	21.7
	12	85	AA	ND	9 (4+5)	6.4
**5**, 1X10^12^vp	13	70	AA	T1c	6 (3+3)	5.9
	14	78	W	T1c		29
	15	72	AA	T1c	7 (3+4)	4

^a^W, white; AA, African American, AsA, Asian American.

^b^ND, not detected.

^c^pre-T PSA is the prostate serum antigen before the initiation of the gene therapy.

### Toxicities

By the study endpoint (day 30), a total of 115 adverse events (AEs) were documented. Ninety-two percent of the AEs were grade 1 (mild) and grade 2 (moderate). Several of treatment-related AEs could be either attributed to Ad5-IL-12 adenovirus (flu-like symptoms, chills, fatigue, altered liver enzyme AST/ALT ratio) or the prodrugs such as diarrhea, anemia, nausea, lymphopenia, leukopenia; Table 2). Only 9 out of 115 (7.8%) adverse events were grade 3. These included leukopenia, lymphopenia, neutropenia and hyponatremia. There was no grade 4 or grade 5 toxicities. Both the incidence, and severity of these events were similar to our four earlier trials in prostate cancer which employed replication competent Ad5 vectors [5,8,9,14]. Due to absence of any DLT or SAE in this trial, we conclude that local administration of replication-competent adenovirus-mediated IL-12 gene therapy is safe in human subjects who present with recurrent localized prostate adenocarcinoma after definitive radiation therapy.

**Table 2:** Adverse events in all cohorts in Ad5-IL-12 gene therapy trial.

Adverse Event by Method of Collection										
	Grade									
	1		2		3		4		Total	
**Physician Assessment**	n	%	n	%	n	%	n	%	n	%
Chills	6	40	1	7	0	0	0	0	7	47
Cytokine release syndrome	0	0	0	0	0	0	0	0	0	0
Mental status change	1	7	0	0	0	0	0	0	1	7
Dehydration	2	13	0	0	0	0	0	0	2	13
Diarrhea	4	27	0	0	0	0	0	0	4	27
Dyspnea	2	13	0	0	0	0	0	0	2	13
Fatigue	6	40	1	7	0	0	0	0	7	47
Fever	5	33	0	0	0	0	0	0	5	33
Flu-like symptoms	3	20	0	0	0	0	0	0	3	20
Hypotension	0	0	0	0	0	0	0	0	0	0
Nausea	4	27	0	0	0	0	0	0	4	27
Urinary frequency	3	20	0	0	0	0	0	0	3	20
Urinary incontinence	0	0	0	0	0	0	0	0	0	0
Urinary retention	0	0	0	0	0	0	0	0	0	0
Urinary tract pain	0	0	0	0	0	0	0	0	0	0
Vomiting	2	13	0	0	0	0	0	0	2	13
Malaise	4	27	0	0	0	0	0	0	4	27
Pain (thigh, kidney, head, fingers)	4	27	0	0	0	0	0	0	4	27
**CBC**										
Anemia	3	20	0	0	0	0	0	0	3	20
Leukopenia	6	40	0	0	2	13	0	0	8	53
Lymphopenia	4	27	3	20	4	27	0	0	11	74
Neutropenia	1	7	0	0	2	13	0	0	3	20
Thrombocytopenia	5	33	0	0	0	0	0	0	5	33
**Blood Chemistries**										
ALT/SGPT increased	4	27	0	0	0	0	0	0	4	27
AST/SGOT increased	2	13	2	13	0	0	0	0	4	26
ALKP increased	1	7	0	0	0	0	0	0	1	7
CPK increased	6	40	2	13	0	0	0	0	8	53
Creatinine increased	6	40	0	0	0	0	0	0	6	40
GGT increased	1	7	0	0	0	0	0	0	1	7
Hypercalcemia	3	20	0	0	0	0	0	0	3	20
Hyperglycemia	0	0	4	27	0	0	0	0	4	27
Hypomagnesemia	1	7	0	0	0	0	0	0	1	7
Hyponatremia	4	27	0	0	1	7	0	0	5	34

All the Adverse events (AEs) collected through primary toxicity endpoint (day 30) are collected and are arranged by method of collection. No adverse events measured for Patient #3 due to consent withdrawal.

### Experimental endpoints

**Fig 3:**
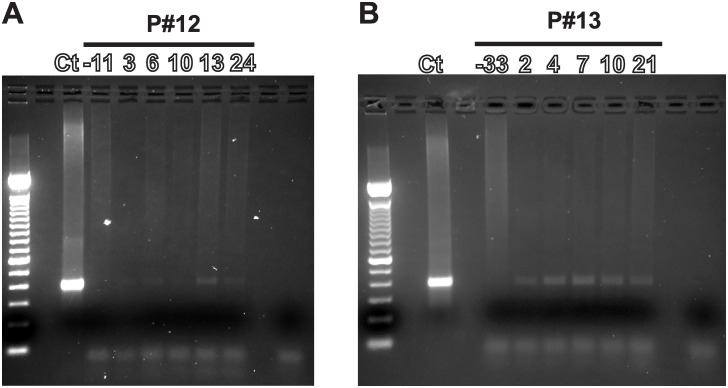
PCR of Ad5-IL-12 adenoviral DNA in blood. Ct, is the positive control. Positive control DNA was isolated blood of a healthy donor spiked with Ad5-yCD/*mut*TK_SR39_*rep*-hIL-12 (1 × 10^4^ vp) adenovirus. Pretreatment blood was drawn up to 30 days (± 3days) prior to study initiation (Ad5-IL-12 adenoviral injection). The numbers above each lane denote the day of each blood draw. Negative control DNA was isolated from the blood from a healthy volunteer with no Ad5-IL-12 DNA added. Agarose gels of the PCR from patient 12 (**A**) and patient 13 (**B**) are shown.

We evaluated the persistence of the adenoviral DNA in patient’s blood as a measure of the viral replication. Presence of Ad5-IL-12 adenoviral DNA detected in patient blood 7–10 days after adenoviral administration suggests potential viral replication (at the site of injection). The viral load was measured by PCR for a target sequence that is common for all our adenoviral vector constructs. We evaluated Ad5-IL-12 adenoviral DNA in all the patients as described in methods. Genomic DNA extracted from the blood donated by a healthy volunteer and spiked with the adenovirus was used as a positive control for the PCR. Viral DNA was not detected in cohorts where patients were injected with low viral dose (cohorts 1–3, 1x10^10^-1x10^11^ vp). Adenoviral DNA was detected only in one patient each in cohort 4 (3x10^11^ vp) and cohort 5 (1x10^12^ vp). Viral DNA in patient #12 (cohort 4) was detected for up to 24 days while it was observed for only 21 days in patient #13 (cohort 5; Fig 3).

### Cytokine response

We measured the levels of IL-12 in patient’s blood after Ad5-IL-12 adenoviral injection. Since IL-12 secretion causes IFN-γ production by NK, NKT, activated CD4+ Th and CD8+ Tc cells and subsequently CXCL10 by monocytes, fibroblasts, and endothelial cells, the expression levels of IFNγ and CXCL10 were also measured. The blood was drawn before viral injection, on the day of the adenoviral injection and on prescribed days up to 42 days and cytokines were quantitated by serum ELISA. Similar to our earlier observations [19], IL-12 could not be measured from serum in first cohort subjects (Table 3). Low level of IL-12 was detected in one patient in cohort 2 (patient #6; 3.7 pg/mL) and in two patients in cohort 3 (1.7 and 1.5 pg/mL). Moderate level IL-12 was detected in two patients in cohort 4 while moderate to high IL-12 was observed in all the three patients in cohort 5 that received the highest dose of the adenovirus (patients # 13–15; 1X10^12^ vp). Peak serum IL-12 was observed on Day 2 post injection in most patients. IFNγ was detected in all the patients except one patient (Table 3).

**Table 3:** Peak cytokine day and concentration.

Cohort	Patient #	IL12		IFNγ		CXCL10	
		Day	pg/mL	Day	pg/mL	Day	pg/mL
1	1		not detected	13	11	28	786.6
	2		not detected	11	37.1		not detected
	3	patient withdrew from the trial on Day 3					
2	4		not detected	2	9	10	192.1
	5		not detected	3	6.7		not detected
	6	2	3.7	2	66.1	7	1567.7
3	7		not detected		not detected		not detected
	8	2	1.7	2	18.7	4	1513.9
	9	4	1.5	2	77.2	9	1854.4
4	10		not detected	2	45.4		552.6
	11	2	31.3	2	24.4	2	1041.8
	12	3	7.2	3	60.1	3	8533.5
5	13	2	33.4	2	705.8	2	9928.6
	14	2	80.9	2	886.9	2	5746.9
	15	2	19.2	2	124.2	2	5119.3

**Fig 4:**
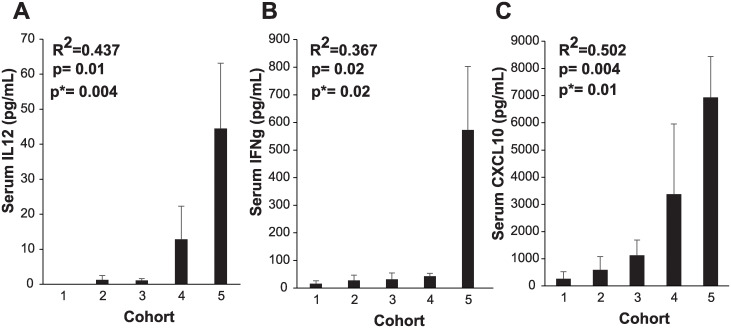
Changes in serum IL-12, IFNγ, and CXCL10 levels in each cohort. Patient blood was drawn within 30 days of the Ad5-IL-12 adenoviral injection or on the day of adenovirus injection prior to the procedure (day 0) and on the days as described in the clinical protocol (up to day 24). Cytokines were measured by ELISA based on cytokine specific standard curves (IL-12 0–500 pg/mL; CXCL10 and IFNγ 0–1,000 pg/mL). The peak cytokine value for each patient was estimated (pg/mL; as presented in Table 3). A mathematical mean value for each cytokine for all the patients within a cohort was estimated and plotted using MS Excel. The mean serum IL-12 (**A**), IFN*γ* (**B**), and CXCL10 (**C**) with errors (standard error of the mean; SEM) is shown for each cohort along with goodness of fit (R2) and significance (p). P values from Jonckheere-Terpstra test is also provided (p*).

**Fig 5:**
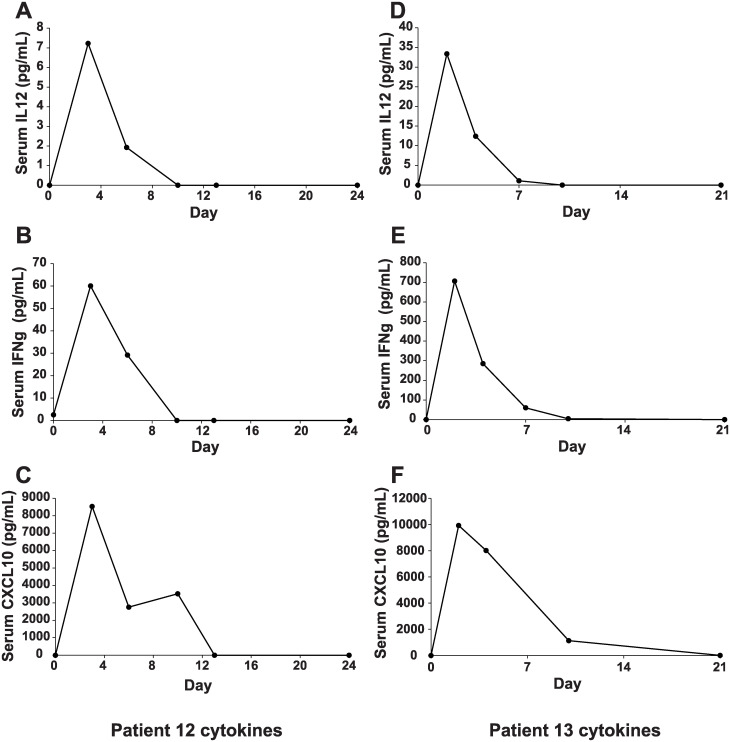
Changes in serum IL-12, IFNγ, and CXCL10 in subjects # 12 and 13. Cytokines were measured by ELISA based on cytokine specific standard curves (IL-12 0–500 pg/mL; CXCL10 and IFNγ 0–1,000 pg/mL). Panels **A-C** show serum IL-12, IFNγ and CXCL10 for patient #12 while panels **D-F** are for patient #13.

Mean serum cytokines, IL-12, IFN-γ and CXCL10, from patients grouped by cohort showed a general trend that patients exhibited increased serum cytokine levels with higher virus doses (Table 3 and Fig 4). Linear regression yielded R^2^ (goodness of fit) of 0.437, 0.367 and 0.502 and p = 0.01, 0.02 and 0.004 for IL12, IFNγ and CXCL10 further supporting that cytokine increase correlated with the viral dose administered. The IFNγ serum levels were unexpectedly high for patients in cohort 5; the correlation coefficient between IFNγ serum level and viral dose improved to 0.985 when IFNγ serum levels from cohorts 1 through 4 alone were considered. CXCL10 was detected in only one patient in cohort 1, and two patients each in cohorts 2 and 3. The highest increase in serum CXCL10 was observed in patient #12 (8533.5 pg/mL) and patient #13 (9928.6 pg/mL), both of which did show detectable levels of the Ad5-IL-12 adenoviral DNA in the blood (Fig 3). Serum IL-12, IFNγ and CXCL10 for the pretreatment and treatment period was plotted for these two patients that demonstrated peak cytokine increased 2-days post Ad5-IL-12 injection (Fig 5). None of these cytokines were detected in the serum before the treatment.

### Shift in CD4^+^, CD8^+^ and NK cells in patient blood

**Fig 6:**
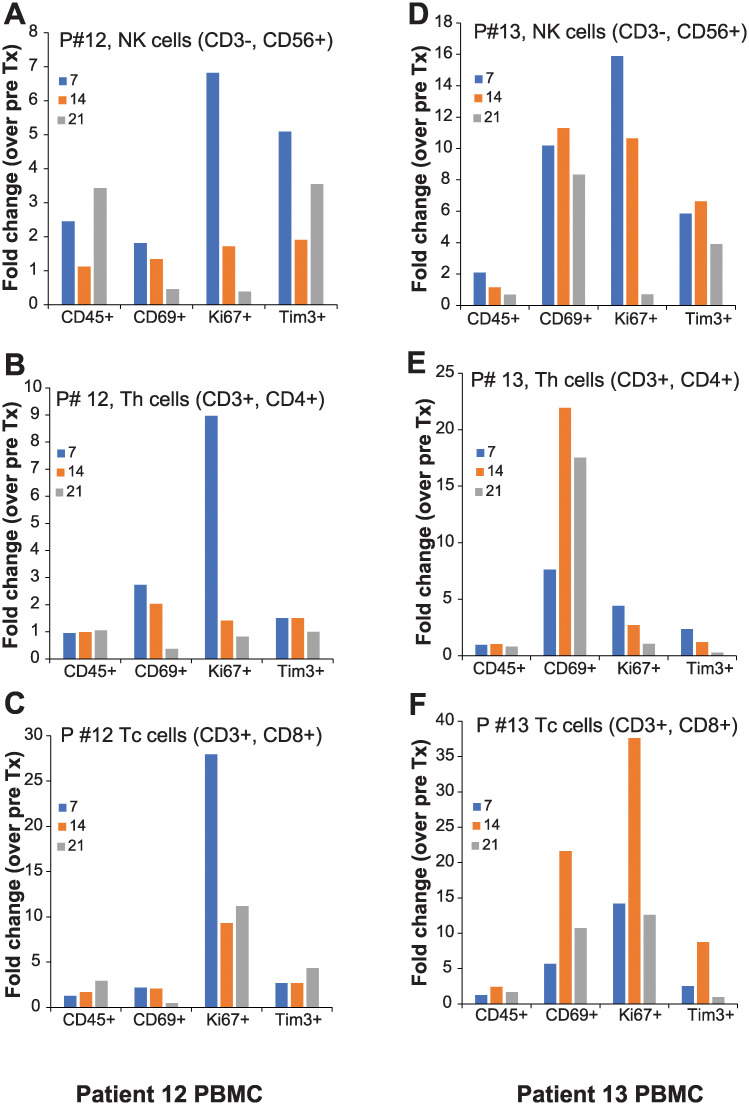
Peripheral blood mononuclear cell (PBMC) counts and markers for patient # 12 and 13. Blood was drawn before and after adenovirus injection on indicated days. Blood was processed, PBMCs were harvested, stained and quantitated by flow-cytometry as described in Materials and Methods. All the data are plotted as mean fold-increase over pre-adenoviral injection (baseline). Natural killer cells (NK), T-helper cells (Th), and cytotoxic T cells (Tc) values from patient #12 are shown in panels **A-C** and patient #13 are shown in panels **D-F**.

The effect of the Ad5-IL-12 gene therapy on immune system activation was evaluated by analyzing circulating PBMCs by flow cytometry. Patient blood was obtained at least once before the Ad5-IL-12 gene therapy administration (baseline) and on follow-up days as described in the clinical protocol. Blood was processed on the day of draw and collected PBMC’s were frozen at -80C until all the samples were collected from a patient. Subsequently, frozen PBMCs were thawed, processed and quantitated by multi-color flow cytometry as described in Materials and Methods. Antibodies against CD3, CD56, CD4, and CD8 served to measure NK, Th and Tc cells while activation of the immune system was monitored by staining for CD45RO, CD69, and proliferation by Ki67. Antibodies against Tim3 were used to detect suppression of the immune system following treatment [21,22]. The data were reported as fold increase compared to the level measured at the pretreatment blood draw for each patient. The data from patient #12 and #13 was presented in Fig 6. We observed nearly 7-fold increase in CD3^−^CD56^+^ proliferating NK cells (Ki67^+^) in patient 12 (Fig 6A) and 10-fold increase in patient 13 (Fig 6D) on day 7 post Ad5-IL-12 gene therapy administration. In comparison, the increase in the CD3^−^CD56^+^Ki67^+^ in cohorts 1–3 was below 5-fold (S1A, S1D and S1G Fig) while it was between 8- and 16-fold in cohorts 4–5 (S1J and S1M Fig) on day 7. In cohort 1, the 7- and 14-day post adenoviral injection data is from patient #1 and #2 only as patient #3 withdrew the consent. In cohort 1, the highest increase was observed 21 days post adenoviral injection (2.5-fold; S1A Fig) while in cohort 5 the highest increase was observed with 7 days post adenoviral injection (16-fold; S1M Fig) which reduced to almost baseline by Day 21. Similar trend was also observed in other cohorts (S1D, S1G and S1J Fig). This data suggests that the time for NK cell maturation was inversely correlated with viral dose.

Similarly, the highest number of Ki67 expressing CD3^+^CD4^+^ T helper (Th) cells was day 21 in cohort 1 (S1B Fig) while in cohort 5 the highest number was observed on day 7 (S1N Fig). In patient 12 and 13 the highest increase in CD3^+^CD4^+^ Ki67^+^ Th cells was also observed on day 7 post adenoviral injection (Fig 6B and 6E). CD3^+^CD8^+^ Tc cells expressing Ki67 also showed maximum increase on day 21 (cohort 1, 3.5-fold; S1C Fig) or day 14 (cohort 5, 30-fold; S1O Fig). In patient 12, the highest increase was observed 7 days post adenoviral injection while in patient 13 it was observed 14 days post injection (Fig 6C and 6F). These changes in Ki67 expression are consistent with the observation that Ad5-IL-12 adenovirus injection in prostatic tumors activated the immune system. Additionally, we observed an increase in the number of cells expressing CD69 (Fig 6 and S1 Fig) which is also a marker for immune system activation [23]. The increase in CD69^+^ cells was apparent in cohort 5 patients which were injected with the highest dose of the adenovirus (CD3^−^CD56^+^CD69^+^ 8-fold; CD3^+^CD4^+^CD69^+^ 14-fold, CD3^+^CD8^+^CD69^+^ 15-fold; S1M, S1N and S1O Fig). The shift in CD69 expressing cells was remarkable compared to cohorts 1–3 (S1A–S1I Fig; less than 2 folds). CD45RO expression was linked to activated CD3^+^CD8^+^ T cytolytic memory cells. Consistent with our earlier observations [19], in cohort 5 we observed an increase in CD45RO expressing CD3^+^CD8^+^ T cells which decreased by day 21 (from approximately 5-fold on day 7 to approximately 2.5-fold on day 21; S1O Fig). In contrast, the fold change in CD3^+^CD8^+^CD45^+^ cells in cohort 1–3 were 1–1.5-fold throughout the study (S1C, S1F and S1I Fig). This data emulated the “On-Off-On” model for memory T cells [24,25] and further supports our earlier findings using the same adenovirus in metastatic pancreatic cancer patients [19].

### Change in PSA levels

**Fig 7:**
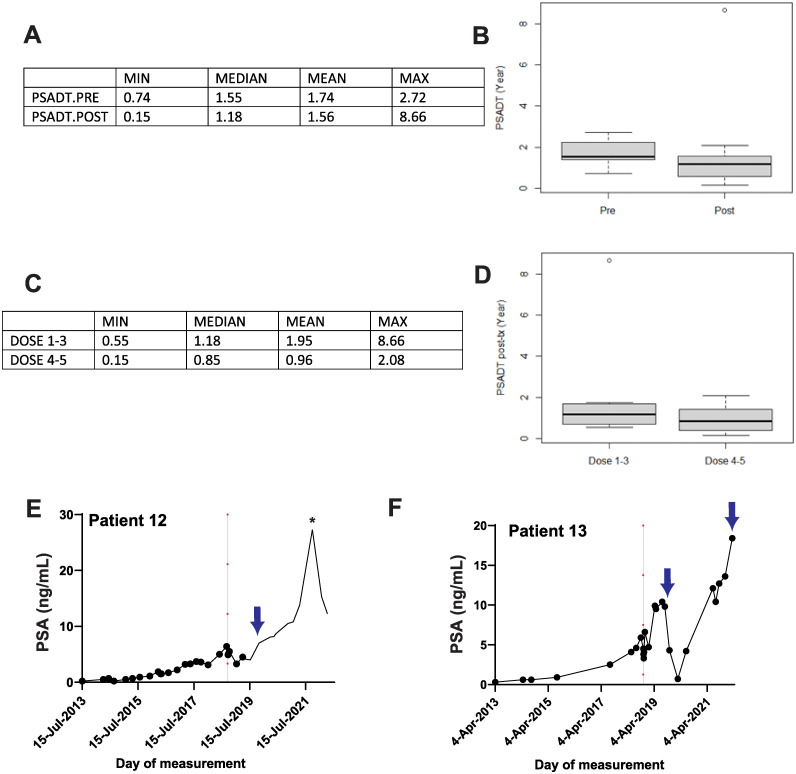
Change in patient PSA in response to gene therapy. PSA doubling time (PSADT) for all the patients were estimated before gene therapy (PSADT.PRE) and after adenoviral injections (PSADT.POST). Mean, median, minimum, and maximum PSADT are tabulated (**A**; years). Median and IQR of pre- and post-gene therapy PSADT is plotted in **B**. Statistical mean, median, minimum, and maximum PSADT for patients that were injected with low-dose of adenovirus (cohorts #1–3) and from patients from high-dose (cohorts #4–5) were estimated and plotted in **C**. A plot of mean, median, minimum, maximum and IQR for both the groups is shown to the right (**D**). Absolute PSA count (pg/mL) for patient #12 (**E**) and #13 (**F**) over time is plotted. Red dotted line indicates the day of Ad5-IL-12 adenoviral injection. Blue arrows indicate date of androgen suppression therapy (AST).

PSA levels were measured from the patients before and after the virus injection. Eight of 15 (53.3%) subjects exhibited ≥20% reduction in PSA just after the initiation of gene therapy (Table 4). The reduction in PSA was short-lived (<6 months) and subsequently all patients relapsed (Fig 7 and S2 Fig). Eleven of the 14 patients have been administered salvage AST (Table 4, Fig 7 and S2 Fig). The median PSA at the initiation of salvage AST was 15 ng/mL. There was 48 months median interval between the initiation of salvage AST and gene therapy (Kaplan–Meier estimation). Patient 12 received single injection of Eligard (leuprolide) in July 2019 and was subsequently diagnosed with lung cancer. This patient was treated with Docetaxel following which he had a surprising decline in PSA (Fig 7E, **asterisk**). Patient 13 received just one dose of AST (Eligard) in October 2019 and restarted in March 2022 (Fig 7F).

**Table 4:** PSA through primary toxicity endpoint (day 30).

Patient	Cohort	Ad dose (vp)	PSA FU (month)	% PSA change	T to PreTx (month)	T to 50% Rise (month)	PSA at Salvage AST (ng/ml)	Delay in additional prostate ca therapy (month)
1	1	1 x 10^10^	9.1	-12.5%	NR	NR		25
2			41.3	-21.4%	31.4	NR		32
3					NE	NE		#
4	2	3 x 10^10^	17.3	0.0%	0.0	5.9	9.3	17
5			39.4	-13.2%	3.0	11.8	10	17
6			37.3	-40.0%	12.0	26.3	27.7	35
7	3	1 x 10^11^	29.8	-36.1%	7.9	24.5	11.2	56
8			22.0	-27.3%	9.0	11.3	106.2	18
9			24.4	-57.7%	NR	NR	24.9	49
10	4	3 x 10^11^	15.9	-6.5%	12.1	15.0	6.4	32
11			13.6	-14.7%	8.4	8.4	126.4	42
12			13.7	-48.4%	13.7	NR	27.3	10
13	5	1 x 10^12^	12.0	-44.1%	0.7	5.4		40
14			8.2	-12.4%	1.4	1.4	203.8	>47
15			8.9	-62.5%	8.9	NR	6.4	15

Ad dose: Adenoviral dose, PSA FU: PSA follow up, T to PreTx: Time (month) before the start of androgen suppression therapy, % PSA decline: PSA decline for each patient within 30 days of oncolytic adenoviral injection; NR: Not reported, NE: Not estimated, AST: Androgen suppression therapy, #: Patient 3 withdrew the consent, although his PSA was still monitored for clinical care it is not reported here.

There was no significant effect of adenovirus injection on the PSA doubling time (PSADT) in individual patients (Fig 7A and 7B). The median pretreatment PSADT was estimated to be 1.55 years compared with 1.18 years post-adenoviral treatment (Fig 7A and 7B). Also, there was no significant effect of adenoviral dose (low dose: cohorts 1–3 versus high dose cohorts 4–5) on PSADT (Fig 7C and 7D). The post-treatment median PSADT was 1.18 years and 0.85 years, respectively. Exemplary PSA (ng/mL) kinetics from individual patients is shown for patients 12 and 13 in Fig 7E and 7F, respectively. PSA as a function of time is shown for remaining patients in S2 Fig. The day of adenoviral injection is indicated by a red vertical line while blue arrows denote the day salvage AST was initiated.

## Discussion

The goal of this phase-1 clinical study was to estimate the maximum tolerated dose of oncolytic, cytotoxic Ad5-IL-12 gene therapy in men with recurrent prostate adenocarcinoma. Patients were administered increasing doses of Ad5-IL-12, ranging from 1 × 10^10^ to 1 × 10^12^ viral particles. Starting on day 3 after adenovirus injection, subjects received 7 days of 5-FC and vGCV prodrugs. Similar to previous studies conducted with equivalent oncolytic adenoviral vectors, majority (>90%) of the AEs in this study were mild to moderate (grade 1/2) that did not involve any medical intervention. No serious adverse events (SAEs) were encountered in the present study indicating that the maximum tolerated dose was not attained. This trial establishes that intra-prostatic injection of Ad5-IL-12 followed by 5-FC and vGCV prodrug therapy is safe.

Investigators from our institution have successfully carried out five phase-I and a phase I/II clinical study utilizing ‘in-house’ developed replication competent, oncolytic adenoviruses in newly diagnosed and locally recurrent prostate cancer [5,6,8,9,11,14]. Additionally, colleagues conducted a phase I trial in locally advanced pancreatic cancer combining the Ad5-yCD/mutTK_SR39_*rep*-ADP virus with gemcitabine and described that it was safe, well tolerated with a median PFS of 11.4 months [26]. We have completed a phase I clinical trial using the same adenoviral vector in metastatic pancreatic cancer where we demonstrated that patients tolerated the Ad5-IL-12 with prodrugs and later received chemotherapy [19]. This trial demonstrated that Ad5-IL-12 adenovirus was safe and well tolerated by patients with metastatic pancreatic cancer. MTD was not reached in the study. Additionally, administration of Ad5-IL-12 caused systemic activation of the immune system as observed by increased serum cytokines (IL-12, IFNγ, and CXCL10), and CD4+, CD8+ T-cells [19]. Also, a clinically meaningful median overall survival benefit (18.4 months, cohort 3 patients vs 4.2 months, cohorts 1 and 2 patients) was observed [20], although this trial was not designed to measure efficacy and the small patient sample size may not provide the statistical power necessary to demonstrate efficacy. A future phase I/II study using a continuous reassessment method (CRM) will be well suited to accurately estimate treatment efficacy.

Akin to the findings in the pancreatic cancer trial that was conducted with the same adenoviral vector [19], in this study involving recurrent prostate cancer we observed similar trends in cytokine concentrations i.e., increase in serum IL-12, IFNγ and CXCL10 with increased adenoviral doses (Fig 4). This data supports the assertion that IL-12 dose provided by a local intratumoral injection elicits a systemic response in patients. Additionally, we observed that the highest increases in cytokines (Fig 5) in patients where Ad5-IL-12 DNA was detected in blood (Fig 3) suggesting that the IL-12 adenovirus was replicating in patients that received a local virus injection and this potentially led to a more potent immune response.

IL-12 is known to mediate the interaction between innate and adaptive immunity [18,27,28]. It activates major components of both the innate immunity (NK and NKT cells) and the adaptive immunity (CD4^+^, CD8^+^ T-cells). Since IL-12 plays multiple roles including priming and increasing T cell survival, promoting differentiation of Th1 cells, and enhancing T, NK, and NKT functions, we quantified the changes in different repertoire of immune cells by quantitating PBMC in blood drawn from patients before and after Ad5-IL-12 adenoviral injection. We observed a trend in NK cell populations (CD3-, CD56+, Ki67+) and adenoviral dose (S1A, S1D, S1G, S1J and S1M Fig). Furthermore, the highest increase in NK cell activity was observed on day 7 in cohorts 2–5, further suggesting that effect may be due to the IL-12 gene therapy. Similarly, increases in the T helper cells (CD3^+^, CD4^+^, Ki67^+^; S1B, S1E, S1H, S1K and S1N Fig) and cytotoxic T cells (CD3^+^, CD8^+^, Ki67^+^; S1C, S1F, S1I, S1L and S1O Fig) correlated with adenoviral dose delivered. Together our data is consistent with a local intratumoral injection of Ad5-IL-12 producing a systemic increase in innate and adaptive immunity resulted from the local administration of IL-12 gene therapy. Our observations are in congruence with published results from a phase I/II trial that used Ad-IL–12 gene therapy for hormone refractory prostate cancer and observed increases in cytokines and immunocompetent cell populations 7–14 days after vector injections in higher dose cohorts [29].

Serum PSA levels and PSA doubling time (PSADT) are key markers of prostate cancer. We followed PSA in patients before and after gene therapy and estimated PSADT. Although, we observed an initial decrease in PSA in 8 out of 14 patients (>20% decrease; Table 4), PSA levels increased during follow up (Fig 7E and 7F and S2 Fig). Also, there was no delay in PSADT when estimated for individual patients (Fig 7A and 7B) or when pooled into low (cohorts 1–3) and high adenoviral dose cohorts (cohorts 4–5; Fig 7C and 7D). This data indicates that although Ad5-IL-12 adenoviral gene therapy was well tolerated by prostate cancer patients, demonstrated a trend toward dose dependency in serum cytokine production and PBMC population, the biological effect perhaps was not sufficient to elicit a significant increase in prostate cancer cell killing. Since the MTD was not reached in this study, we speculate that future phase I/II trials that include a dose-escalation regime (with continued reassessment method; CRM) may have utility in safely increasing the IL-12 dose and could be used to estimate the efficacy of increased viral load in combination with cytotoxic prodrugs.

Earlier trials failed to deliver on the promise of a potent proinflammatory cytokine due to toxicities associated with its systemic delivery. Localized IL-12 delivery provides enhanced spatiotemporal distribution, generates systemic antitumor immunity from a locally initiated immune response, brings rapid increase in pro-inflammatory cytokines, including IFN-γ, and IL-6, and reduces opposing negative signaling [30]. Various methods have been developed to deliver IL-12 to patients including recombinant cytokine, plasmid-based, virus-based, cell-based, and m-RNA based delivery. Here, we employed an engineered, replication-competent, lytic adenovirus (Ad5-IL-12) that not only delivers two different suicide genes along with IL-12, but also selectively replicates in cancer cells to deliver locally high concentrations. Absence of reaching the MTD in this trial supports the safety of our approach of using an in-house developed adenoviral vaccine and directly injecting into the prostate tumors; the lack of toxicities confirms the work of others using Ad-IL-12 adenovirus [31]. In light of the safe approach of others have repeatedly (up to 3 times) injected adenoviral vaccines intratumorally [31,32], multiple injections should be a consideration in future trials. Fujita et al., [33] delivered Ad-mIL-12 vaccine along with radiotherapy in metastatic prostate cancer preclinical model and observed that the combination of Ad-mIL-12 with radiotherapy was better at controlling tumor over Ad-mIL-12 monotherapy or control vector. In this study we did not find significant changes in serum PSA or PSADT, and we speculate that combining with cytotoxic agents such as radiotherapy may be more beneficial than the monotherapy (employed in this trial). In this regard, the results of ongoing clinical trials that combine IL-12 with cytotoxic chemotherapy (NCT04633252) or AST (NCT05361798) in prostate cancer patients is highly anticipated.

## Materials and methods

### Study design

The study was designed as a single site, prospective, non-randomized, phase I, classical 3+3 dose escalation trial of viral particles (vp). This trial was designed to enroll 15–30 men with locally recurrent prostate cancer after definitive radiotherapy under 5 cohorts with increasing doses. Each cohort of three patients with clinically localized recurrent prostate adenocarcinoma were intraprostatically injected with increasing doses (cohort-1 1x10^10^ vp, cohort-2 3x10^10^ vp, cohort-3 1x10^11^ vp, cohort-4 3x10^11^ vp, and cohort-5 1x10^12^ vp) of the Ad5-yCD/*mut*TK_SR39_*rep*-hIL-12 (Ad5-IL-12) adenovirus followed by one week (7 days) of 5-fluorocytosine and valganciclovir prodrug therapy. All subjects were treated in the Department of Radiation Oncology and the Department of Urology in the Henry Ford Cancer Institute, Henry Ford Health.

The primary endpoint of this investigator-initiated phase-I study was to determine the dose-dependent toxicity and maximum tolerated dose (MTD) of oncolytic adenovirus-mediated cytotoxic and IL-12 gene therapy in men with locally recurrent prostate cancer after definitive radiotherapy. Secondarily we evaluated PSA response including PSA doubling time (PSADT) before and after administration of the study therapy. The exploratory aim was to determine a possible association between the primary and secondary outcomes and immunological endpoints including serum IL-12 and IFN-γ levels, and NK cell cytolytic activity. The protocol received all required regulatory approvals before initiation and was conducted under BB-IND 16536 (IRB-9829; Clinical Trials # NCT02555397). Good clinical practices were used throughout.

### Patient selection and eligibility criteria

Patients were required to have biopsy proven local recurrence of prostate cancer at least one year after the completion of definitive radiation therapy. The biopsy must have been performed within 180 days of study registration. Serum PSA < 100 ng/ or evidence of biologically active disease as demonstrated by an unequivocally rising serum PSA level that is ≥ 2 ng/mL above the nadir. Subjects were eligible if at least 18 years old with Karnofsky performance status ≥70. Patients were required to have negative lymph nodes as established by imaging (pelvic CT or pelvic MRI) within 90 days of registration. Men with lymph nodes equivocal or questionable by imaging were eligible if the nodes were ≤ 1.0 cm. Men with positive lymph nodes by capromab pendetide (ProstaScint) scans were eligible provided a corresponding lymph node identified by CT or MR imaging was ≤ 1.0 cm. Patients were required to demonstrate no evidence of metastatic disease, as evaluated by a bone scan and CT scan of the abdomen and pelvis within 90 days prior to registration. Equivocal bone scan findings were allowed if plain films were negative for metastasis. Patients were required to have adequate baseline organ function, as assessed by the following laboratory values, before initiating the protocol, including: (i) adequate renal function with serum creatinine ≤1.5 mg/dl or creatinine clearance ≥50 ml/min/m2; (ii) platelet count >100,000/μL; (iii) absolute neutrophil count >1,000/μL; (iv) hemoglobin >10.0 g/dL; and (v) bilirubin <1.5 mg/dL, and serum glutamic oxaloacetic transaminase and serum glutamic pyruvic transaminase <3 times the upper limit of normal. Patients had to have the ability to give informed consent and express a willingness to meet all of the expected requirements of the protocol for the duration of the study.

Patients with any one of the following conditions were excluded from the study: (i) PSA ≥ 100 ng/mL, (ii) prostate volume > 100 cc, (iii) pathologically positive lymph nodes or nodes > 1.0 cm on imaging (nodes > 1.0 cm but biopsy negative were allowed), (iv) evidence of M1 metastatic disease, (v) prior invasive malignancy except for non-melanoma skin cancer within 5 years of enrollment. Subjects were required to be disease-free for > 5 years, (vi) prior radical prostatectomy, cryosurgery for prostate cancer, or bilateral orchiectomy for any reason, (vii) biochemical failure while on AST if the subject had prior AST, (viii) prior systemic chemotherapy for prostate cancer. Prior chemotherapy for a different cancer was allowed given patients were >2 years post-completion of chemotherapy at the time of registration. Subjects on Proscar therapy were required to stop Proscar therapy to be eligible, (ix) severe active co-morbidity such as New York Heart Association Class II or greater congestive heart failure or active ventricular arrhythmia requiring medication, Chronic obstructive pulmonary disease (COPD) exacerbation or other respiratory illness requiring hospitalization within last 3 months or precluding study therapy at the time of registration, (x) acute infection, (xi) previous history of livery diseases including hepatitis, (xii) immunosuppressive therapy including systemic corticosteroids, and (xiii) impaired immunity or susceptibility to serious viral infections. All patients met the eligibility requirements of the protocol and signed the informed consent document. There were no protocol violations. All patients were recruited between October 2015 to March 2019.1.

### Design and manufacturing Ad5-yCD/*mut*TK_SR39_*rep*-hIL-12 adenovirus

The design and construction of the replication-competent Ad5-yCD/*mut*TK_SR39_*rep*-hIL-12 (Ad5-IL-12) adenovirus has been described previously [19]. Clinical-grade (GMP) adenovirus was manufactured at the Baylor College of Medicine Gene Vector Laboratory (Houston, TX). The adenovirus was supplied as a sterile, clear, frozen liquid in vials containing 1.25 ml at a concentration of 1.0 × 10^12^ vp/ml. The vp-to-pfu ratio of the undiluted final product was 15. The potency of the clinical lot was re-evaluated every 6–12 months and there was no reduction in potency during the study.

### Pretreatment planning and injection of Ad5-yCD/*mut*TK_SR39_*rep*-hIL-12 adenovirus

Informed consent was obtained from all patients before study-specific procedures were initiated. Pretreatment planning included transrectal ultrasound guided needle biopsy of the prostate and computerized tomography (CT) simulation. Transrectal ultrasound scan (TRUS)-guided biopsy cores (≥ 6 cores) were taken to map the location of the cancer within the prostate. Blood was collected for baseline detection of the Ad5-IL-12 adenovirus by polymerase chain reaction (PCR) and serum cytokines (IL-12, IFNγ and CXCL10) by Enzyme-Linked Immunosorbent Assay (ELISA).

Men received a single intraprostatic injection of Ad5-IL-12 adenovirus on day 1. The injection was performed on an outpatient basis as described previously [5,6,8–10,34]. The total injection volume was 3.0 mL. The adenovirus was distributed over the entire prostate; however, the dose distribution was skewed to sextants with cancer, based on the baseline biopsy report (Table 1). Two days later, prodrugs (5FC and vGCV) were administered as described below. Patient compliance was monitored by the study nurse. AST was not allowed until biochemical recurrence was documented.

### Prodrug administration

Prodrugs were administered on an outpatient basis. 5-Fluorocytosine (Ancobon; Roche Laboratories, Basel, Switzerland) was administered orally beginning on day 3 and continued for 1 week (7 days). A total of 150 mg/kg/day was given in four equally divided doses. Valganciclovir (Valcyte; Roche Laboratories) was administered orally beginning on day 3 and continued for 1 week (7 days). A total of 1,800 mg/day was given in two equally divided doses every 12 h. The research nurse assigned to the trial counted the pills periodically to monitor patient compliance.

### Patient monitoring

Toxicity assessments were performed once a week prior to the start of chemotherapy (first 3 weeks) and then at scheduled follow-up visits at 3, 6, 9, 12, 18, and 24 months. Toxicities were graded using the National Cancer Institute’s Common Toxicity Criteria, CTCAE v.4.03. Case report forms (CRFs) specifically designed for this trial were used to document adverse events (AEs). The study was monitored by an internal Data and Safety Monitoring Board (DSMB). The primary end point, treatment toxicity, was monitored up to and including day 30. After treatment, patients received standard urologic care.

The following evaluations were conducted once a week following the adenovirus injection through day 30: (i) physical examination, (ii) blood chemistries and complete blood counts including peripheral blood mononuclear cells (PBMC) (iii) serum PSA, (iv) presence of Ad5-IL-12 viral DNA and infectious adenovirus in blood, (v) cytokines including IL-12, CXCL10 and IFNγ, and (vi) performance status. The presence of Ad5-IL-12 viral DNA in blood, serum IL-12, IFNγ, and CXCL10 levels were monitored at every blood draw until not detected in two consecutive measurements.

### PCR of Ad5-yCD/*mut*TK_SR39_*rep*-hIL-12 adenoviral DNA blood

Blood was obtained before the adenovirus injection and at least once a week after for semi-quantitative determination of adenoviral DNA in blood. Total genomic DNA was isolated from 2.5–3 mL of blood using Qiagen columns following the protocols recommended by the manufacturer (QIAGEN GmbH, Hilden, Germany). 2–3 μL of eluted DNA was used as a template for the PCR reactions. The PCR primers have been previously described [8]. The 5′ primer hybridizes to the linker between the yCD and mutant HSV-1 TK gene, and the 3′ primer hybridizes to the mutant HSV-1 TK gene. The PCR product is 388 bp in length and specific for the yCD/mutTK_SR39_ fusion gene contained in Ad5-IL-12. To generate a standard curve, 2.5 × 10^9^ vp of Ad5-IL-12 was added to 0.5 ml of human volunteer blood making a concentration of 5.0 × 10^9^ vp/ml. Serial 10-fold dilutions were prepared down to 50 vp/ml using human volunteer blood as the diluent. The sensitivity of the assay was 50 vp/mL.

### Serum cytokines

IL-12, IFNγ and CXCL12 were measured in patient serum. Blood was drawn once pretreatment (between days −30 and −1) and then again on days +3, +7, +14, +21 and +28 (±3 days) to isolate serum and collect PBMCs. ELISA was performed following manufacturer’s protocol (BD Biosciences). For measuring CXCL10, serum samples were run after 10-fold and 100-fold dilution. Undiluted serum was used for IL-12 and IFNγ measurement. Log range dilution of purified cytokines were used for generating standard curve. Absorbance was measured at 450 nm from 96-well ELISA plates. The raw data was organized using MS Excel and analyzed on GraphPad Prism version 9. The data was interpolated using a standard curve with a second order polynomial (quadratic) equation and plotted with a 95% confidence interval (CI). The correlation coefficient (R^2^) values were >0.98 for all the analyses. Total cytokine levels were estimated for each patient for every blood draw. The highest increase in the cytokine levels (peak) after adenoviral injection and the day of “peak” serum cytokine was estimated for each patient (Table 3). The peak serum cytokine values for IL-12, IFNγ and CXCL12 for all the patients within a cohort were used to detect any adenoviral dose dependent trends in cytokine levels using R with linear regression.

### Flow cytometry

Flow cytometry was performed as previously described [19]. PBMCs were isolated from the blood drawn at different times as described above. PBMCs were quickly thawed, washed, fixed in FACSLyse solution (BD Biosciences, San Jose, CA) after lysing and removing RBCs. Cells were pelleted, blocked in FC block (BioLegend, San Diego, CA) and were incubated with a cocktail of anti-human antibodies including CD3 conjugated with allophycocyanin-Cy7 (APC-Cy7), CD56 conjugated with PEDazzle 594, CD45RO conjugated with R-phycoerythrin-Cy7 (PE-Cy7), CD8 conjugated with BV510, CD69 conjugated with PE, Tim3 conjugated with BV421 (BioLegend), and CD4 conjugated with peridinin chlorophyll protein Cy5.5 (PerCP-Cy5.5,BD Biosciences). Following incubation, the cells were washed, fixed (Fix/Perm buffer; eBioscience, Waltham, MA) and incubated with Ki67 conjugated with APC (BioLegend) or mouse IgG1 k isotype control antibody (BioLegend), washed, resuspended in wash buffer and sorted on a LSRFortessa (BD Biosciences). One-hundred thousand gated events were collected for each sample and data was analyzed using BD FACSDiva software V.8.0.1 (BD Biosciences).

### Statistical methods

Adverse events, graded using CTCAE v4.03, were collected through day 30 (day 1 being the day of the adenovirus injection). The effect of oncolytic adenovirus-mediated cytotoxic and IL-12 gene therapy on serum PSA levels was evaluated by estimating PSA doubling time (PSADT). PSADT was estimated for each patient pre-injection and post-injection. A single model of log (PSA) on time was built for each patient, and the PSA doubling time was calculated for each patient for pre-injection PSADT. For post-injection PSADT estimation, PSA doubling time from nadir was estimated using all PSA values. Minimum (MIN), Median and Mean values were estimated for pre- and post- injection PSADT. Additionally, post-injected PSADT for cohorts 1–3 and 4–5 were pooled.

PSADT was calculated assuming first-order kinetics using the following equation:

$$\text{Ln}\left(\text{PSA}\right)=\beta \text{o}+\beta 1\text{t}$$

where βo is the point where the regression line crosses the ordinate at t = 0 and β1 is the number of units that Ln (PSA) changes for every one-unit change in time (i.e., the slope). PSADT is defined as Ln (2) / β1. Jonckheere-Terpstra test was performed to identify trend for the increases in serum cytokine levels.

## Supporting information

S1 Fig An estimation of peripheral blood mononuclear cells (PBMCs) for all cohorts. (DOCX)

S2 Fig Serum PSA counts in all subjects over time. (DOCX)

S1 File Phase 1 trial of oncolytic adenovirus-mediated cytotoxic and interleukin 12 gene therapy for locally recurrent prostate cancer after definitive radiotherapy. (PDF)

S2 File Request for planned change(s). (PDF)
