# Supplementary material for: A phase I clinical trial of oncolytic adenovirus mediated suicide and interleukin-12 gene therapy in patients with recurrent localized prostate adenocarcinoma
Source: PLoS One. 2023 Sep 15;18(9):e0291315. doi: 10.1371/journal.pone.0291315 (PMC10503775; doi:10.1371/journal.pone.0291315)
Supplement: S1 Fig — (DOCX) [file pone.0291315.s001.docx]

**S1** **Fig. An estimation of peripheral blood mononuclear cell (PBMC) for all cohorts.** Blood was before and after Ad-5-Il-12 adenoviral injection. Blood samples were processed and PBMC were counted as described in Materials and Methods. All data are reported as mean fold-increase over baseline blood collected prior to adenovirus injection for three patients within each cohort. In cohort 1, the 7- and 14-day post adenoviral injection data is from patient #1 and #2 only as patient #3 withdrew the consent. No error bar on day-21 data point in cohort-1 is shown because only one sample was analyzed at this time point. Natural killer cells (NK; CD3^-^, CD56^+^), T-helper cells (Th; CD3^+^, CD4^+^), and cytotoxic T cells (Tc; CD3^+^ CD8^+^) fold change values are shown with standard deviation for cohorts #1-5.
