# Supplementary material for: A phase I clinical trial of oncolytic adenovirus mediated suicide and interleukin-12 gene therapy in patients with recurrent localized prostate adenocarcinoma
Source: PLoS One. 2023 Sep 15;18(9):e0291315. doi: 10.1371/journal.pone.0291315 (PMC10503775; doi:10.1371/journal.pone.0291315)
Supplement: S2 Fig — (DOCX) [file pone.0291315.s002.docx]

**S2 Fig.** **Serum PSA counts in all subjects over time**. Absolute PSA values (ng/mL) are plotted on Y-axis while time is on the X-axis. The red dotted lines indicate the day of Ad5-yCD/mutTKSR39rep-hIL-12 adenoviral injection. The serum PSA follow-up time varied from 0.3 yrs (patient 8) to 6 yrs (patient 5). A sharp drop in the PSA level indicates the time when the salvage androgen suppression therapy (AST) was initiated. PSA for Patient #3 is not presented due to consent withdrawal while patients #12 and #13 PSA values are provided in **Figs 6E and F**.
