# Supplementary material for: A phase I clinical trial of oncolytic adenovirus mediated suicide and interleukin-12 gene therapy in patients with recurrent localized prostate adenocarcinoma
Source: PLoS One. 2023 Sep 15;18(9):e0291315. doi: 10.1371/journal.pone.0291315 (PMC10503775; doi:10.1371/journal.pone.0291315)
Supplement: S1 File — (PDF) [file pone.0291315.s003.pdf]

PHASE 1 TRIAL OF ONCOLYTIC ADENOVIRUS-MEDIATED CYTOTOXIC AND INTERLEUKIN 12 GENE THERAPY FOR LOCALLY RECURRENT PROSTATE CANCER AFTER DEFINITIVE RADIOTHERAPY

Principal Investigator:	Hans Stricker, MD[1]
Co-Principal Investigators:	Mohamed Elshaikh, MD[2]<br>Farzan Siddiqui, MD, PhD[2]<br>Haythem Ali, MD[3]<br>Clara Hwang, MD[3]<br>Benjamin Movsas, MD[2]<br>James Peabody, MD[1]<br>Svend O Freytag, PhD[2]
Institutions:	Vattikuti Urology Institute[1]<br>Department of Radiation Oncology[2]<br>Department of Internal Medicine[3]<br>Henry Ford Health Systems<br>Detroit, MI 48202

version 7; revised 02/16/2016

TABLE OF CONTENTS

		Page
	Face Page	1
	Table of Contents	2
	Eligibility Checklist	3 - 5
1.0	Background	6 - 18
2.0	Objectives	19
3.0	Subject Selection	19 - 21
4.0	Registration Procedures	22
5.0	Study Therapy and Protocol	22 - 29
6.0	Subject Assessments	30 - 36
7.0	Data Collection	36 - 39
8.0	Statistical Considerations	39 - 41
9.0	Data and Safety Monitoring Plan	41
10.0	Inclusion of Minorities	42
11.0	Process of Informed Consent	42
12.0	Literature Cited	43 - 46

Eligibility Checklist

Subject Study Number: ___ ___ ___	Subject Initials: ___ ___ ___

Eligibility Checklist – Please refer to primary source documents for this information.

Questions 1 - 29 must all be answered Yes for the subject to be eligible to participate.

1. The subject has biopsy-proven adenocarcinoma of the prostate at least 1 year after completion of definitive radiation therapy and within 180 days of registration? 0[ ] No 1[ ] Yes

   When was the biopsy performed? ___ ___ / ___ ___ / ___ ___ ___ ___
   M M D D Y Y Y Y

   When was the last day of definitive radiation therapy? ___ ___ / ___ ___ / ___ ___ ___ ___
   M M D D Y Y Y Y

2. The subject has evidence of biologically active disease as demonstrated by an unequivocally rising serum PSA level that is ≥ 2 ng/mL above the nadir? 0[ ] No 1[ ] Yes

   Last PSA value: ___ ___ . ___ ng/mL assessed on ___ ___ / ___ ___ / ___ ___ ___ ___
   M M D D Y Y Y Y

   PSA nadir: ___ ___ . ___ ng/mL assessed on ___ ___ / ___ ___ / ___ ___ ___ ___
   M M D D Y Y Y Y

3. The last PSA is < 100 ng/mL? 0[ ] No 1[ ] Yes

4. The subject is ≥ 18 years of age? 0[ ] No 1[ ] Yes

   Age: ___ ___ years

5. The subject has a Karnofsky performance status ≥ 70?. 0[ ] No 1[ ] Yes

   Karnofsky performance status ___ ___

6. The subject has negative lymph nodes within 90 days of registration? 0[ ] No 1[ ] Yes

7. The subject has no evidence of metastatic disease within 90 days of registration? 0[ ] No 1[ ] Yes

8. The subject has adequate renal function with serum creatinine ≤ 1.5 mg/dL or creatinine clearance > 50 mL/min/m^2? 0[ ] No 1[ ] Yes

   Serum creatinine: ___ . ___ mg/dL or Creatinine clearance: ___ ___ . ___ mL/min/m^2

9. The subject has a platelet count > 100,000/µL (100 K/µL)? 0[ ] No 1[ ] Yes

   Platelet count: ___ ___ ___ K/µL

Eligibility Checklist

Subject Study Number: ___ ___ ___ Subject Initials: ___ ___ ___

10. The subject has an absolute neutrophil count > 1,000/µL(1 K/µL)? 0[ ] No 1[ ] Yes

    Neutrophil count: ___ ___ . ___ K/µL

11. The subject has a hemoglobin > 10.0 g/dL? 0[ ] No 1[ ] Yes

    Hemoglobin: ___ ___ . ___ g/dL

12. The subject has a bilirubin < 1.5 mg/dL? 0[ ] No 1[ ] Yes

    Bilirubin: ___ . ___ mg/dL

13. The subject has a SGOT ≤ 3.0 x ULN? 0[ ] No 1[ ] Yes

    SGOT: ___ ___ . ___ IU/L

14. The subject has a SGPT ≤ 3.0 x ULN? 0[ ] No 1[ ] Yes

    SGPT: ___ ___ . ___ IU/L

15. The subject is willing to use effective contraception while on treatment and for at least 3 months afterwards? 0[ ] No 1[ ] Yes

16. The subject's prostate volume is not > 100 cc? 0[ ] No 1[ ] Yes

    Prostate volume: ___ ___ . ___ cc

17. The subject has not had a prior invasive malignancy except for non-melanoma skin cancer within the past 5 years? 0[ ] No 1[ ] Yes

18. The subject has not had prior radical prostatectomy, cryosurgery or bilateral orchiectomy? 0[ ] No 1[ ] Yes

19. If the subject had prior androgen deprivation therapy (ADT), the subject did not exhibit biochemical (PSA) failure while on ADT? 0[ ] No 1[ ] Yes

20. The subject has not had prior chemotherapy for the study cancer? 0[ ] No 1[ ] Yes

21. Major surgery is not planned within 3 months of registration? 0[ ] No 1[ ] Yes

22. The subject does not have any of the following co-morbidities? 0[ ] No 1[ ] Yes

    -New York Heart Association Class II or greater congestive heart failure or active ventricular arrhythmia requiring medication.
    -Chronic obstructive pulmonary disease (COPD) exacerbation or other respiratory illness requiring hospitalization within last 3 months or precluding study therapy at the time of registration.
    -Acute infection. Acute infection is defined by any viral, bacterial, or fungal infection that requires specific therapy within 72 hours of initiation of the study therapy.

Eligibility Checklist

Subject Study Number: ___ ___ ___ Subject Initials: ___ ___ ___

23. The subject does not have a previous history of liver disease including hepatitis? 0[ ] No 1[ ] Yes

24. The subject is not taking immunosuppressive therapy including systemic corticosteroids? 0[ ] No 1[ ] Yes
    Note: Use of inhaled and topical corticosteroids is permitted.

25. The subject does not have impaired immunity or susceptibility to viral infections? 0[ ] No 1[ ] Yes

26. The subject does not have a known allergy to any product used in the protocol? (If the subject has an allergy to Ciproflaxin, another antibiotic can be substituted at the discretion of the treating physician). 0[ ] No 1[ ] Yes

27. The subject has no serious medical or psychiatric illness or concomitant medication, which, in the judgment of the investigator, might interfere with the subject's ability to respond to or tolerate the treatment or complete the trial? 0[ ] No 1[ ] Yes

28. The subject has the ability to give informed consent and has expressed a willingness to meet all of the expected requirements of the protocol for the duration of the study? 0[ ] No 1[ ] Yes

29. The subject gave informed consent? 0[ ] No 1[ ] Yes

    Date the Informed Consent Form was signed: ___ ___ / ___ ___ / ___ ___ ___ ___
    M M D D Y Y Y Y

Completed By Initials: ___ ___ ___

Form Completion Date: ___ ___ / ___ ___ / ___ ___ ___ ___
M M D D Y Y Y Y

1.0 BACKGROUND

An estimated 221,000 men will be diagnosed with prostate cancer in the United States in 2015 and approximately one-third will elect radiation therapy (external beam and/or brachytherapy) as their primary treatment (1). For men with organ-confined, low-risk disease (Stage T1/T2, Gleason ≤ 6, PSA < 10 ng/mL), radiation therapy results in excellent long-term survival and disease control (2). However, it is less effective against more aggressive forms of the disease (Stage ≥ T3, Gleason ≥ 7, PSA > 10 ng/mL) and a significant fraction of patients develop disease recurrence within 10 years. Fortunately, the rate of occult distal failure following definitive radiotherapy is < 10% at 10 years (3). Hence, there is a substantial window of opportunity after initial recurrence in which the disease may still be largely localized and amenable to local salvage therapies.

Men with locally recurrent prostate cancer after definitive radiotherapy have few therapeutic options that have a high likelihood of eradicating the tumor with a reasonable degree of safety. Other than expectant management and systemic therapy (i.e., androgen deprivation therapy, ADT), there are four available local treatment options for locally recurrent prostate cancer: 1) salvage radical prostatectomy (RP), 2) salvage cryoablation (CA), 3) salvage brachytherapy (BT), and 4) salvage high-intensity focused ultrasound (HIFU). Although some have demonstrated encouraging 5-year disease-free survival rates in single institution trials, most are associated with significant morbidity and there are no prospective randomized studies demonstrating their long-term effectiveness.

1.1 Local Salvage Therapies for Radio-Recurrent Prostate Cancer

1.1.1 Radical Prostatectomy

Salvage RP following definitive radiotherapy has resulted in disease-free survival of 55% - 71% at 5 years and 30% - 43% at 10 years (4, 5). Patients with organ-confined disease, who represent 20% - 50% of cases, have an excellent prognosis. However, the complication rate associated with salvage RP is high. Urinary incontinence is observed in 20% - 68% of patients, anastomotic stricture in 9% - 32%, and erectile dysfunction in over 72%.

1.1.2 Cryoablation

Recent technical innovations have led to a re-emergence in the interest in CA as a minimally invasive alternative to salvage RP. In patients treated with third-generation cryotechnology, 5-year disease-free survival is 44% - 73% (5). Incontinence is observed in 4% - 40% of cases, obstruction/retention in 3% - 21%, and erectile dysfunction in over 72%. Hence, the results with salvage CA are comparable to salvage RP.

1.1.3 Brachytherapy

Five-year disease-free survival following salvage BT is 34% - 87% (4, 5), although many patients also received short-term hormone therapy confounding the results. As expected, most of the complications associated with salvage BT were genitourinary (GU) or gastrointestinal (GI) in nature. Grade 3/4 GU toxicity (incontinence, frequency/urgency, hesitancy, nocturia) is 14% - 47% and grade 3/4 GI toxicity (rectal ulcers, bleeding) is 0% - 24%.

Only one study reported erectile dysfunction, which was 100% grade 2.

1.1.4 HIFU

There have been only three published reports on salvage HIFU and the follow-up is relatively short (< 3 years). The largest study (167 patients) reported 3-year disease-free survival of 53%, although 18% of patients also received hormone therapy. Complications associated with salvage HIFU include incontinence (7% - 50%), urinary obstruction/retention (9% - 36%), urinary stricture (17% - 20%), recto-urethral fistula (3% - 7%). Only one study reported erectile dysfunction, which was 72%.

1.2 Oncolytic Adenovirus-Mediated Cytotoxic Gene Therapy (OAMCGT)

1.2.1 Preclinical Studies

Suicide gene therapy is an investigational cancer therapy that is currently being evaluated in clinical trials (6). It is based on the premise that cycling tumor cells expressing the suicide gene will be rendered sensitive to specific pharmacologic agents thereby providing a therapeutic index. Delivery of the suicide gene to the tumor is usually accomplished by direct intratumoral or systemic injection of a viral vector containing the suicide gene.

Two suicide genes that have been evaluated in preclinical models and in the clinic are the *E. Coli* cytosine deaminase (CD) and herpes simplex virus thymidine kinase (HSV-1 TK), which confer sensitivity to 5-fluorocytosine (5-FC) and ganciclovir (GCV), respectively (7-25). The mechanism of CD/5-FC killing is mediated through its ability to inhibit thymidylate synthase (TS) resulting in the depletion of dTMP, which is required for DNA synthesis. The mechanism of HSV-1 TK/GCV killing is mediated through its ability to inhibit DNA chain elongation. Both the CD/5-FC and HSV-1 TK/GCV suicide gene systems exhibit a bystander effect, which results in the destruction of neighboring tumor cells not expressing the suicide genes (7-9).

The concept of using CD and HSV-1 TK suicide gene therapies to improve the effectiveness of EBRT was first proposed by Kim *et al*. (26-28). The product of the CD/5-FC reaction, 5-FU, is a well established chemotherapeutic and radiosensitizer in the clinic. The mechanism of 5-FU radiosensitization is believed to be mediated through the inhibition of TS (by 5-FdUMP), resulting in the depletion of dTMP pools and increased DNA strand breaks as well as redistribution of cells in early S phase, a radiosensitive phase of the cell cycle. It was hypothesized that the HSV-1 TK/GCV system may also result in tumor cell radiosensitization by preventing the repair of radiation-induced DNA damage. Both suicide gene systems demonstrated marked tumor cell radiosensitization *in vitro* resulting in sensitization enhancement ratios (SER) in the range of 1.8 to 2.2 (26-28).

Because the CD/5-FC system increases DNA strand breaks and the HSV-1 TK/GCV system may inhibit the repair of those breaks, Rogulski *et al*. were the first to propose combining the two suicide gene systems (29). They generated a novel CD/HSV-1 TK fusion gene that could confer both 5-FC

and GCV sensitivity onto cells. Implementation of both suicide gene systems simultaneously without, or with, EBRT proved to be far superior than either system alone *in vitro* and *in vivo* (29-32).

To further enhance the effectiveness of CD and HSV-1 TK suicide gene therapies in combination with EBRT, Freytag *et al*. generated a replication-competent oncolytic adenovirus (Ad5-CD/TK*rep*) expressing the prototype CD/HSV-1 TK fusion gene (33). Not only do replication-competent adenoviruses provide a therapeutic effect via their cytolytic activity (oncolytic viral therapy), they also result in a greater efficiency of gene transduction *in vivo* relative to replication-defective adenoviruses. Moreover, the multiplicative property of replication-competent adenoviruses results in a greater therapeutic gene copy number per infected cell as well as a greater number of tumor cells infected due to local viral spread. The merit of oncolytic replication-competent adenovirus-mediated suicide gene therapy in combination with EBRT (trimodal therapy) has been evaluated in several preclinical tumor models (34, 35). In an orthotopic prostate tumor model, oncolytic adenovirus-mediated cytotoxic gene therapy (OAMCGT) significantly improved both local and metastatic tumor control relative to EBRT alone (35).

1.2.2 Clinical Trials

The toxicity and efficacy of OAMCGT has been evaluated in five clinical trials of prostate cancer (36-44), including a prospective randomized phase 2 study (**Table 1**).

Table 1. Gene Therapy Trials Conducted by the Sponsor

Trial	# Subjects	Oncolytic Adenovirus	Phase	Indication[1]	Radiation[2]	Median FU[3]
1	16	Ad5-CD/TK*rep*	1	LRPC	N	12.9 yrs
2	15	Ad5-CD/TK*rep*	1	NDPC	Y	11.5 yrs
3	9	Ad5-yCD/*mut*TK$_{SR39}$*rep*-ADP	1	NDPC	Y	8.2 yrs
4	18	Ad5-yCD/*mut*TK$_{SR39}$*rep*-hNIS	1	NDPC	Y	6.8 yrs
5	44	Ad5-yCD/*mut*TK$_{SR39}$*rep*-ADP	2	NDPC	Y	5.2 yrs

[1]LRPC, locally recurrent prostate cancer; NDPC, newly diagnosed prostate cancer.
[2]N, no; Y, yes.
[3]Median PSA follow-up of surviving subjects as of 05/15/15. Subjects lost to follow-up were excluded.

Three different (but related) oncolytic adenoviruses armed with therapeutic genes have been used:

1) Trials 1 & 2- used the first-generation Ad5-CD/TK*rep* adenovirus, which contains a bacterial CD (bCD)/wild-type HSV-1 TK fusion gene (bCD/HSV-1 TK). Trial 1 enrolled 16 subjects with locally recurrent prostate cancer (36, 38). Trial 2 enrolled 15 subjects with newly diagnosed intermediate- to high-risk prostate cancer and included radiation therapy (37).

2) Trials 3 & 5- used the second-generation Ad5-yCD/*mut*TK$_{SR39}$*rep*-ADP adenovirus, which contains an improved yeast CD (yCD)/mutant HSV-1 TK$_{SR39}$ fusion gene (yCD/*mut*TK$_{SR39}$) and the adenovirus death protein

(ADP) gene (45). Both the yCD and mutant HSV-1 TK_{SR39} genes have better catalytic properties than the bCD and wild-type HSV-1 TK genes contained in Ad5-CD/TK*rep* (46, 47). ADP increases the cytolytic activity of replication-competent adenoviruses (48, 49). Trial 3 enrolled 9 subjects with newly diagnosed intermediate- to high-risk prostate cancer and included radiation therapy (39). Trial 5 was a prospective randomized phase 2 study that compared the toxicity and efficacy of combining OAMCGT with contemporary dose (80 Gy) intensity modulated radiotherapy (IMRT) versus IMRT alone and enrolled 44 subjects with intermediate-risk prostate cancer (44).

3) <u>Trial 4</u>- used the second-generation Ad5-yCD/*mut*TK_{SR39}*rep*-hNIS adenovirus, which contains the improved yCD/*mut*TK_{SR39} fusion gene and the human sodium iodide symporter (hNIS) reporter gene. hNIS allows for non-invasive imaging of recombinant adenoviruses using single photon emission computer tomography (SPECT) or positron emission tomography (PET) when coupled with radioactive tracers such as sodium pertechnetate ($Na^{99m}TcO_4$) or iodide (^{123}I, ^{125}I or ^{131}I), respectively (50, 51). This trial enrolled 18 subjects with newly diagnosed intermediate- to high-risk prostate cancer and included radiation therapy (42, 43).

The results of trial 1 (phase 1 study in locally recurrent setting), trial 5 (randomized phase 2 study in newly diagnosed setting), and trial 4 (phase 1 study in newly diagnosed setting with non-invasive imaging) are described.

1.2.2.1 Locally Recurrent Prostate Cancer (Trial 1)

1.2.2.1.1 Description

Sixteen subjects with locally recurrent prostate cancer after definitive radiotherapy were enrolled at the Henry Ford Health System between January 2000 and December 2001 (36). All men received a single intraprostatic injection of the first-generation Ad5-CD/TK*rep* adenovirus on day 1. The adenovirus dose was escalated from 1 x 10^{10} vp (cohort 1) to 1 x 10^{12} vp (cohorts 3 & 4) in four cohorts. 5-FC + GCV prodrug therapy was administered for one (cohorts 1 - 3) and 2 (cohort 4) weeks.

The primary endpoint was acute toxicity through day 30. Secondary endpoints included PSA response, transgene expression and tumor destruction in post-treatment biopsies, adenoviral DNA in blood, infectious adenovirus in blood and urine, and neutralizing antibodies (NAB) to adenovirus.

1.2.2.1.2 Short-term Results

There were no dose-limiting toxicities (DLTs) or serious adverse events (SAEs). Ninety-four percent of the AEs observed were mild or moderate (grade 1/2) in nature. There were nine (6%) grade 3 events including four events of hyperglycemia, and one event each of cardiac ischemia, dyspnea, hyper-magnesemia, lymphopenia, and neutropenia. All subjects exhibiting grade 3 hyperglycemia were diabetics. The one event of grade 3 cardiac ischemia occurred in a subject with a history of heart disease. The one event of grade 3 dyspnea occurred in a subject with a history of chronic obstructive pulmonary disease. The grade 3 events of hypermagnesemia, lymphopenia, and neutropenia each occurred in a single subject and were transient. There were no grade 4 events.

Seven of 16 (44%) subjects demonstrated a > 25% decrease, and 3 of 16 (19%) demonstrated a > 50% decrease, in serum PSA lasting < 120 days. There was excellent correlation between persistence of the Ad5-CD/TK*rep* adenovirus, as determined by viral DNA in blood, and PSA response. Transgene expression and tumor destruction at the injection site were confirmed by needle biopsy at 2 weeks. Two subjects were negative for adenocarcinoma at 1 year.

Ad5-CD/TK*rep* DNA was detected in blood as far out as day 76. No infectious adenovirus was detected in serum or urine at any time point. All subjects developed high titers of NAB to adenovirus.

1.2.2.1.3 Long-term Results

Long-term outcome was assessed at 5 and 7 years. At 5 years, PSA doubling time (PSADT) and delay in the administration of additional salvage therapy was examined (38). At 7 years, overall (OS) and disease-specific (DSS) survival were compared to well-matched historical controls (41).

When considering all evaluable subjects (n = 14), PSADT increased from a mean of 17 months (prior to gene therapy) to 31 months (after gene therapy) (P = 0.014) (**Table 2**). A greater percentage of subjects who received the highest adenovirus dose exhibited an increase in PSADT suggesting a dose-dependent effect. Assuming that salvage androgen deprivation

therapy (ADT) would have been initiated at a PSA of 15 ng/mL, administration of the gene therapy delayed the projected onset of salvage ADT by a mean of 2.2 years in all evaluable subjects and 2.6 years in subjects receiving the highest adenovirus dose.

Table 2. 5-Year Analysis in Locally Recurrent Setting

	Mean PSADT		Delay in Salvage ADT[2]
**Subject cohort**	**Before GT (months)**	**After GT (months)**	
All evaluable subjects (n = 14)[1]	17	31	26 months (2.2 years)
Highest Ad dose subjects (10^{12} vp; n = 6)	18	32	31 months (2.6 years)
**Subject cohort**	**Subjects who exhibited increase in PSADT**		
Two lowest Ad dose cohorts (10^{10} and 10^{11} vp)	3 of 8 (38%)		
Highest Ad dose cohort (10^{12} vp; n = 6)	5 of 6 (83%)		

[1]PSADT after the gene therapy could not be determined in two subjects owing to a non-treatment-related death, and implementation of salvage ADT 2 months after gene therapy at subject's request.
[2]The delay in administration of salvage ADT was determined by calculating when the PSA would have crossed a threshold of 15 ng/mL using pre-gene therapy versus post-gene therapy PSADT.

OS and DSS were examined at 7 years and compared to well-matched historical controls (**Figure 1** and **Table 3**). The control cohort included 154 patients with locally recurrent prostate cancer treated at the University of Michigan between 1986 and 2000 (52). At 7 years, OS was 69% vs. 44% (P = .07), and DSS was 94% vs. 55% (P = .03), in the gene therapy vs. the control cohort, respectively. Median survival was 8.6 vs. 5.9 years in the gene therapy vs. the control cohort, respectively.

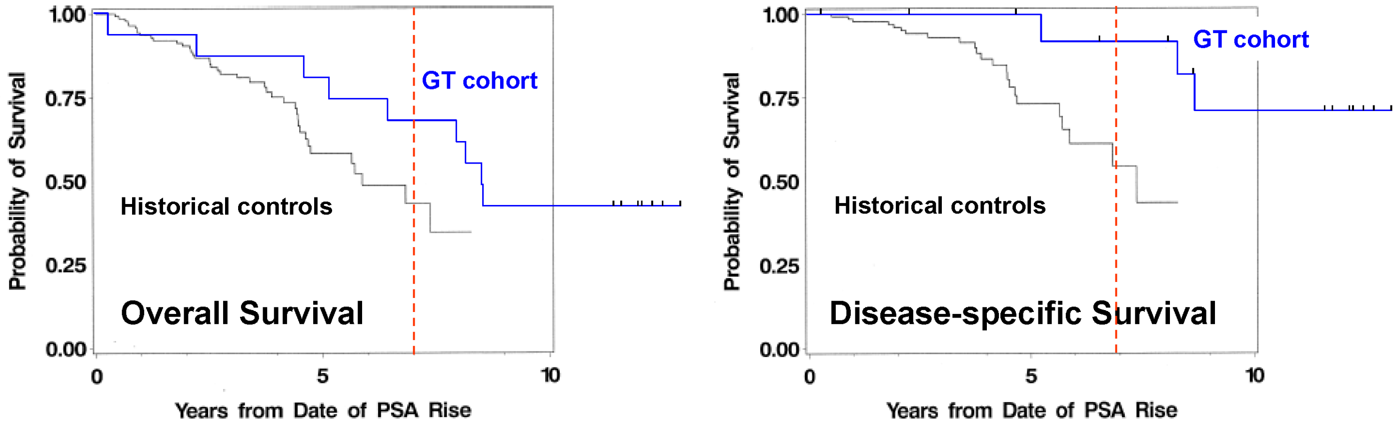

Figure 1. Overall and disease-specific survival. The control cohort included 154 patients with locally recurrent prostate cancer treated at the University of Michigan (52). For the gene therapy cohort, men that expired with evidence/symptoms of metastatic disease or were lost to follow-up were declared a death due to prostate cancer. Ticks marks indicate where subjects were censored. Red dotted line indicates 7 years.

Table 3. Overall and Disease-Specific Survival		
	**Gene Therapy Cohort**	**Control Cohort**
7-year OS	69%	44%
7-year DSS	94%	55%
Median survival (years)	8.6	5.9

1.2.2.1.4 Summary of Results in Locally Recurrent Setting

OAMCGT was associated with minimal toxicity when administered to men with locally recurrent prostate cancer. There was suggestive evidence of long-term benefits as demonstrated by a lengthening of PSADT, a delay in when additional salvage therapy is indicated, and possible improvements in overall and disease-specific survival.

1.2.2.2 Newly Diagnosed Prostate Cancer In Combination With Contemporary Dose IMRT (Trial 5)

1.2.2.2.1 Description

Forty-four men with newly diagnosed intermediate-risk prostate cancer were enrolled at the Henry Ford Health System and Johns Hopkins Medical Institute between January 2008 and July 2010 (44). Subjects were randomly assigned to receive either OAMCGT plus 80 Gy IMRT (arm 1; n = 21) or 80 Gy IMRT only (arm 2; n = 23). Subjects were stratified into three groups based on Gleason score, PSA, and percent positive biopsy cores at baseline. Men in arm 1 received a single intraprostatic injection (1 x 10^{12} vp) of the second-generation Ad5-yCD/*mut*TK$_{SR39}$*rep*-ADP adenovirus on day 1 followed by 2 weeks (weekdays only) of 5-FC + valganciclovir (vGCV) prodrug therapy. IMRT (40 x 2 Gy for 80 Gy) commenced with the initiation of the prodrug therapy. Men in arm 2 received IMRT (40 x 2 Gy for 80 Gy) only. Hormone therapy was not allowed until biochemical recurrence was documented.

The primary phase 2 endpoint was acute (≤ 90 days) toxicity. Secondary endpoints included freedom from biochemical/clinical failure (FFF), prostate biopsy (12 core) positivity at 2 years, disease-specific and overall survival, and quality of life (QOL),

1.2.2.2.2 Results

Men in arm 1 exhibited a greater incidence of flu-like symptoms, transaminitis, neutropenia and

thrombocytopenia relative to men in arm 2. These events were expected and attributable to the oncolytic adenovirus (flu-like symptoms, transaminitis) and prodrug therapy (neutropenia, thrombocytopenia). The vast majority (98%) of AEs were grade 1/2 and transient. Less than 10% of men in each arm exhibited acute (≤ 90 days) ≥ grade 2 GI toxicity. While acute ≥ grade 2 GU events were more prevalent (arm 1- 43%, arm 2- 31%), no significant difference in acute GI or GU toxicity was noted between the two arms. There was no significant difference in QOL between the two treatment arms.

Prostate biopsy outcome ≥ 2 years after radiotherapy is highly prognostic for long-term outcome (53-55). Hence, short-term efficacy was assessed by a 12 core prostate biopsy 2 years after completion of IMRT. Two-year biopsies were obtained on 37 (84%) men. There was a 42% (P = 0.13) and 34% (P = 0.14) relative reduction in biopsy positivity on arm 1 based on actual biopsies and intent-to-treat (ITT), respectively (**Table 4**). This type 1 error is within the accepted range for randomized phase 2 trials (56). There was a 60% (P = 0.07) and 48% (P = 0.08) relative reduction in biopsy positivity on arm 1 in men with < 50% positive biopsy cores at baseline based on actual biopsies and ITT, respectively. Owing to the adenovirus injection algorithm that skewed the adenovirus dose distribution to the positive sextants, men in arm 1 with < 50% positive cores received 2.5 times the adenovirus dose/positive core than men with ≥ 50% positive cores.

To date, 1 subject in arm 1 (4.8%) and 2 subjects in arm 2 (8.7%) have exhibited biochemical failure. No subject has developed hormone-refractory or metastatic disease and none have died from prostate cancer.

Table 4. 2-Year Prostate Biopsy Results				
		Subjects with Positive Biopsy[1]		
		Arm 1	Arm 2	*P* value
**All subjects**	Biopsied	6/18 (33%)	11/19 (58%)	0.13
	ITT[2]	9/21 (43%)	15/23 (65%)	0.14
**Stratification Group[3]**				
1	Biopsied	0/1 (0%)	1/1 (100%)	> 0.99
	ITT	0/1 (0%)	1/1 (100%)	> 0.99
2	Biopsied	3/13 (23%)	8/14 (57%)	0.07
	ITT	5/15 (33%)	11/17 (65%)	0.08
3	Biopsied	3/4 (75%)	2/4 (50%)	0.47
	ITT	4/5 (80%)	3/5 (60%)	0.49
**% Positive biopsy cores**				
< 50%	Biopsied	3/13 (23%)	8/14 (57%)	0.07
	ITT	5/15 (33%)	11/17 (65%)	0.08
≥ 50%	Biopsied	3/5 (60%)	3/5 (60%)	> 0.99
	ITT	4/6 (67%)	4/6 (67%)	> 0.99

[1]Number of subjects with a positive 2-year biopsy over the total number of subjects with percentage in parentheses.

[2]Intent-to-treat (ITT) includes all subjects whether or not a 2-year biopsy was obtained. Those subjects that were not biopsied were given a positive score.

[3]Group 1- Gleason score 5/6 and PSA < 10 ng/mL and ≥ 50% positive biopsy cores. Group 2- (Gleason score 5/6 and PSA 10 - 20 ng/mL) or (Gleason score 7 and PSA 0 - 20 ng/mL) and < 50% positive biopsy cores. Group 3- (Gleason score 5/6 and PSA 10 - 20 ng/mL) or (Gleason score 7 and PSA 0 - 20 ng/mL) and ≥ 50% positive biopsy cores.

1.2.2.2.3 Summary of Results in Newly Diagnosed Setting In Combination With Contemporary Dose IMRT

There was no significant difference in GI/GU events and QOL between the two arms. There was a 42% relative reduction in 2-year biopsy positivity on the gene therapy arm (arm 1). There was a 60% relative reduction in 2-year biopsy positivity on the gene therapy arm in men who had < 50% positive biopsy cores at baseline. The results indicate that combining OAMCGT with contemporary dose IMRT does not exacerbate the most common side effects of prostate radiotherapy and results in a clinically meaningful reduction in positive biopsies at 2 years in men with intermediate-risk prostate cancer.

1.2.2.3 Newly Diagnosed Prostate Cancer In Combination With Conformal Radiotherapy and Non-Invasive Imaging (Trial 4)

1.2.2.3.1 Description

Eighteen men with newly diagnosed intermediate- to high-risk prostate cancer were enrolled at the Henry

Ford Health System between May 2006 and January 2010 (42, 43). All men received a single intraprostatic injection of the second-generation Ad5-yCD/*mut*TK$_{SR39}$*rep*-hNIS adenovirus on day 1. The adenovirus dose was escalated from 1 x 10^{11} vp (cohort 1) to 5 x 10^{12} vp (cohort 3) in three cohorts. 5-FC + vGCV prodrug therapy was administered for 2 weeks (weekdays only). Following the adenovirus injection, subjects were administered $Na^{99m}TcO_4$ on various days and subjected to SPECT imaging.

The primary endpoint was acute toxicity through day 90. Secondary end points included feasibility of using hNIS as a reporter gene to monitor adenoviral gene therapy vectors non-invasively *in vivo*, volume and magnitude of gene expression in the prostate, kinetics and persistence of reporter gene expression, whole body distribution of $^{99m}TcO_4$ uptake, PSA response, and 2-year prostate biopsy positivity.

1.2.2.3.2 Results

hNIS reporter gene expression (i.e., $^{99m}TcO_4$ uptake) was detected in 0 of 3 (0%) subjects at 1 x 10^{11} vp, 6 of 8 (75%) subjects at 1 x 10^{12} vp, and 6 of 6 (100%) subjects at 5 x 10^{12} vp. Gene expression was readily detected in the prostate following injection of 1 x 10^{12} vp and 5 x 10^{12} vp (**Figure 2**). Serial imaging on consecutive days demonstrated that gene expression in the prostate lasted ≤ 7 days (**Figure 3**).

Importantly, whole body planar scans demonstrated that no reporter gene expression was detected outside of the prostate in any subject (**Figure 4**).

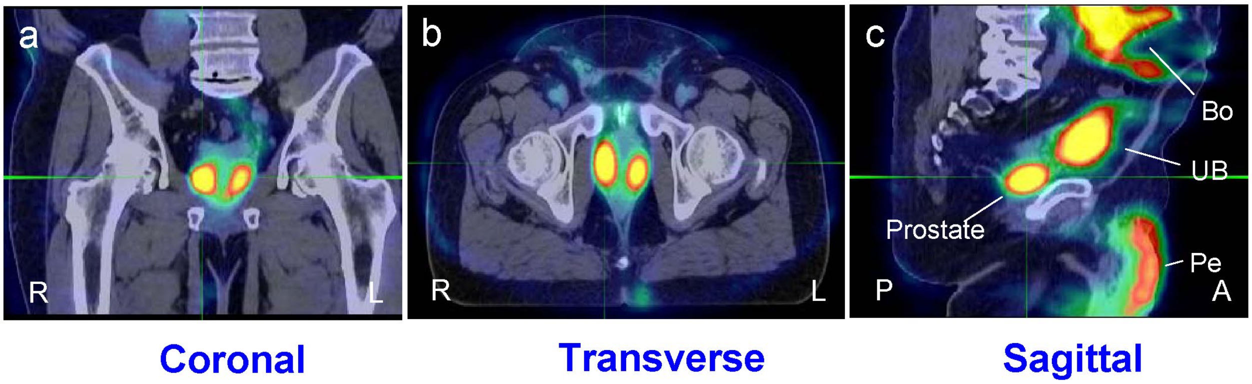

Figure 2. Non-invasive imaging of gene expression in the prostate. The subject received an intraprostatic injection of 5 x 10^{12} vp of the Ad5-yCD/*mut*TK$_{SR39}$*rep*-hNIS adenovirus on day 1. 16 mCi $Na^{99m}TcO_4$ was administered on days 2 and 3 and SPECT was performed 4 hours later. Day 3 images are shown. Radiotracer uptake is indicated by the colors (yellow > red > green). Activity in the urinary bladder (UB) is due to excretion of the radiotracer through that organ. Activity in the penis (Pe) is due to blood flow

through that organ. Activity in the bowel (Bo) is due to trapping. L, left; R, right; P, posterior; A, anterior.

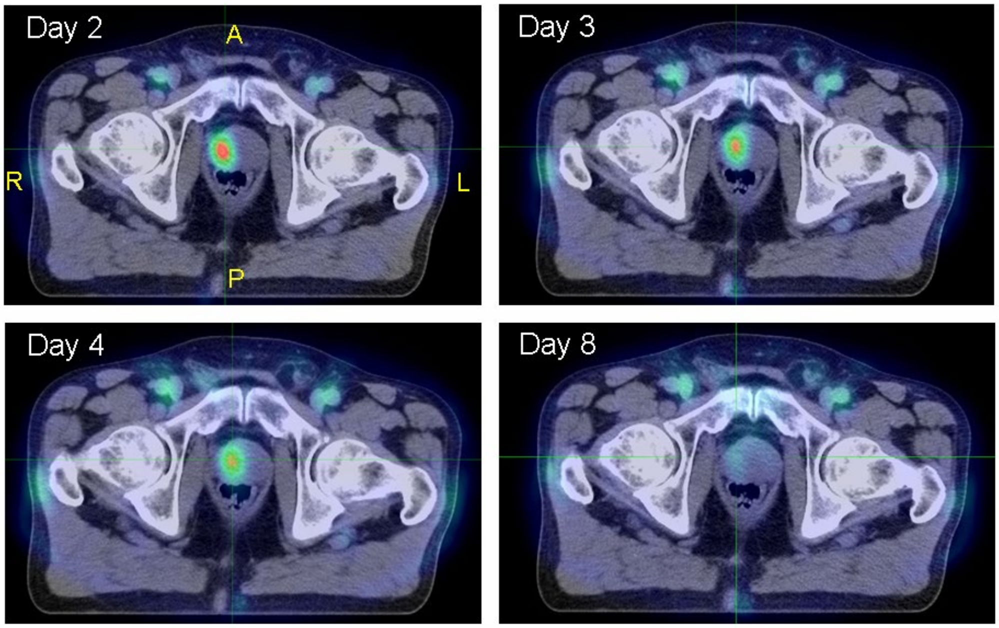

Figure 3. Persistence of gene expression in the prostate. A subject with intermediate-risk prostate cancer received an intraprostatic injection (right side only) of Ad5-yCD/*mut*TK$_{SR39}$*rep*-hNIS (1 x 10^{12} vp) on day 1. $Na^{99m}TcO_4$ (16 mCi) was administered on days 2, 3, 4 and 8 and SPECT imaging (whole body and pelvic planar scans, pelvic SPECT) commenced 4 h later. hNIS gene expression (i.e., $^{99m}TcO_4^-$ uptake) was detected in the right side of the prostate on all days. L, left; R, right; P, posterior; A, anterior.

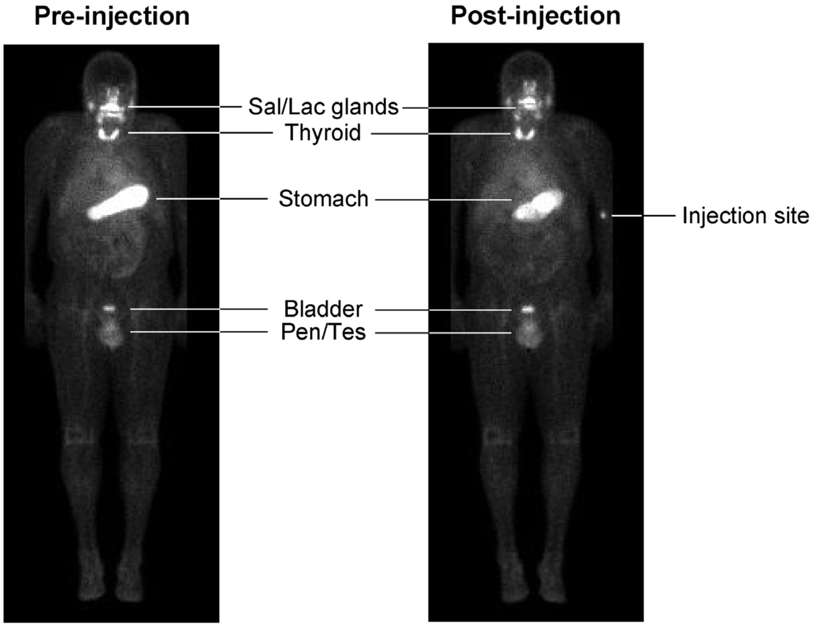

Figure 4. Whole body distribution of $^{99m}TcO_4$ uptake. A subject with intermediate-risk prostate cancer received an intraprostatic injection of Ad5-yCD/*mut*TK$_{SR39}$*rep*-hNIS (1 x 10^{12} vp) on day 1. $Na^{99m}TcO_4$ (16 mCi) was administered on day 4 and SPECT imaging commenced 4 h later. Shown are whole body planar scans prior to (pre-injection) and three days after (post-injection) the adenovirus injection. Activity in the salivary glands (Sal), lachrymal glands (Lac), thyroid gland and stomach are due to endogenous hNIS

expression. $Na^{99m}TcO_4$ is secreted through the urinary tract, accounting for the activity in the urinary bladder. The activity observed in the penis (Pen) and testicles (Tes) is due to blood flow through those structures. The intravenous $^{99m}TcO_4$ injection site can be seen in the post-injection scan.

1.2.2.3.3 Summary of Results in Newly Diagnosed Setting In Combination With Conformal Radiotherapy and Non-Invasive Imaging

Gene expression was readily detected in the prostate at adenovirus doses ≥ 1 x 10^{12} vp. Gene expression peaked on days 2 or 3 and last ≤ 7 days in most subjects as determined by SPECT. No gene expression was detected outside the prostate in any subject. At an adenovirus dose of 5 x 10^{12} vp, on average, 45% of the prostate volume was covered with gene expression (43).

1.3 Interleukin 12 (IL-12) Gene Therapy

To enhance our investigational approach further, we have added IL-12 to our therapeutic platform (57). IL-12 was selected for evaluation for several reasons. IL-12 plays an essential role in the interaction between innate and adaptive immunity (58-60). It is normally produced by phagocytes (monocytes/macrophages, neutrophils) and dendritic cells in response to pathogens, and directly activates cells of the innate (NK and NK-T cells) and adaptive ($CD4^+$ and $CD8^+$ cells) arms of immunity. IL-12 helps to prime T cells and increases their survival, promotes Th1 differentiation, enhances T cell, NK cell, and NK-T cell effector functions, and induces the secretion of IFN-γ, which mediates many of IL-12's anti-tumor effects. IFN-γ acts directly on tumor cells as well as stromal and endothelial cells within the tumor microenvironment. IFN-γ signaling results in 1) increased MHC class I processing and presentation (enhancing recognition of tumor by T cells); 2) induction of chemokines (IP-10 and MIG) that may recruit innate and adaptive immune effectors, which in turn lead to 3) alterations in extracellular matrix remodeling, including inhibition of matrix metalloproteinase expression, that reduces angiogenesis and tumor invasion; and 4) decreased expression of adhesion molecules by endothelial cells that may further limit angiogenesis. Hence, the rationale behind IL-12 immunotherapy lies in its ability to induce IFN-γ expression, promote immune cell survival and activation, enhance productive antigen presentation, and inhibit tumor angiogenesis.

IL-12 has demonstrated significant anti-tumor activity in preclinical models including prostate cancer (61). However, its activity in the clinic has been modest when administered as a recombinant protein or expressed from retrovirally-transduced fibroblasts (59). Furthermore, IL-12 has resulted in severe toxicity including two treatment-related deaths when administered systemically as a recombinant protein (62, 63). We believe that IL-12 produced by a locally administered, oncolytic adenovirus may exhibit better activity in the clinic without excessive toxicity relative to previous approaches. Administering the adenovirus intraprostatically (rather than systemically) should result in a high, local concentration of IL-12 within the prostate. This should limit systemic toxicity and promote IL-12's anti-tumor effects. IL-12 has been well-tolerated in humans when administered locally (59). Moreover, we believe our multi-modal approach may function like an autologous, whole tumor

vaccine. All modalities (*oncolytic viral therapy, cytotoxic gene therapy, IL-12 gene therapy*, and *radiotherapy* when combined with radiation) result in significant tumor cell destruction and the release of tumor antigens. This may facilitate antigen cross-presentation by professional APCs, which, when coupled with IL-12's ability to promote Th1 differentiation, may lead to the development of antigen-specific, cell-mediated immunity.

We have demonstrated in a preclinical model of prostate cancer that the addition of IL-12 to our therapeutic platform improves both local and metastatic tumor control that is mediated by both the innate and adaptive arms of immunity (57).

1.4 Health-Related Quality of Life (HRQOL) and Utilities

1.4.1 Expanded Prostate Index Composite (EPIC)

The Expanded Prostate Cancer Index Composite (EPIC) is a comprehensive instrument designed to evaluate patient function and bother after prostate cancer treatment. EPIC is a robust prostate cancer HRQOL instrument that measures a broad spectrum of urinary, bowel, and sexual symptoms related to radiotherapy (64). The domains (e.g., urinary, bowel, sexual) were validated separately; since each domain will be used intact there is no threat to validity (65).

1.4.2 Utility as Measured by the EQ-5D

The EQ-5D is a two-part, self-assessment questionnaire that takes approximately 5 minutes to complete (66). The first part consists of 5 items covering 5 dimensions including: mobility, self care, usual activities, pain/discomfort, and anxiety/depression. Each dimension has an index score of 1- no problems, 2- moderate problems, or 3- extreme problems. The second part is a visual analogue scale (VAS) valuing current health state, measured on a 20-cm 10-point interval scale. Worst imaginable health state is scored as 0 at the bottom of the scale, and best imaginable health state is scored as 100 at the top. Both the 5-item index score and the VAS score are transformed into a utility score between 0 "Worst health state" and 1 "Best health state". The EQ-5D will be completed by the patient.

2.0 OBJECTIVES

2.1 Primary

The primary purpose of this phase 1 study is to determine the dose-dependent toxicity and maximum tolerated dose (MTD) of oncolytic adenovirus-mediated cytotoxic and IL-12 gene therapy in men with locally recurrent prostate cancer after definitive radiotherapy.

2.2 Secondary

To assess:

2.2.1 PSA response.

2.2.2 Freedom from biochemical/clinical failure (FFF).

2.2.3 PSA doubling time (PSADT) before and after administration of the study therapy.

2.2.4 Disease-specific and overall survival.

2.2.5 Quality of life (QOL).

2.3 Exploratory

2.3.1 Possible association between the primary and secondary outcomes and immunological endpoints including serum IL-12 and IFN-γ levels, and NK cell cytolytic activity.

3.0 SUBJECT SELECTION

3.1 Number of Subjects

Fifteen to 30 men with locally recurrent prostate cancer after definitive radiotherapy.

3.2 Criteria for Subject Eligibility

To be eligible, the subject must have:

3.2.1 Biopsy-proven local recurrence of prostate cancer at least one year after the completion of definitive radiation therapy. The biopsy must be performed within 180 days of registration.

3.2.2 Evidence of biologically active disease as demonstrated by an unequivocally rising serum PSA level that is ≥ 2 ng/mL above the nadir.

3.2.3 PSA < 100 ng/mL.

3.2.4 Age ≥ 18 years

3.2.5 Karnofsky performance status ≥ 70.

3.2.6 Negative lymph nodes as established by imaging (pelvic CT or pelvic MRI) within 90 days of registration.

3.2.6.1 Subjects with lymph nodes equivocal or questionable by imaging are eligible if the nodes are ≤ 1.0 cm.

3.2.6.2 Subjects with positive lymph nodes by capromab pendetide (ProstaScint) scans are eligible provided a corresponding lymph node identified by CT or MR imaging is ≤ 1.0 cm.

3.2.7 No evidence of metastatic disease, as evaluated by bone scan and CT scan of the abdomen and pelvis within 90 days prior to registration. Equivocal bone scan findings are allowed if plain films are negative for metastasis.

3.2.8 Subjects must have adequate baseline organ function, as assessed by the following laboratory values, within 30 days before initiating the study therapy:

3.2.8.1 Adequate renal function with serum creatinine ≤ 1.5 mg/dL or creatinine clearance >50 mL/min/m^2.

3.2.8.2 Platelet count > 100,000/µL.

3.2.8.3 Absolute neutrophil count > 1,000/µL.

3.2.8.4 Hemoglobin > 10.0 g/dL.

3.2.8.5 Bilirubin < 1.5 mg/dL

3.2.8.6 AST/SGOT and ALT/SGPT < 3.0 times upper limit of normal (ULN).

3.2.9 Men of child-producing potential must be willing to consent to use effective contraception for at least 3 months after the gene therapy.

3.2.10 Subjects must possess the ability to give informed consent and express a willingness to meet all of the expected requirements of the protocol for the duration of the study.

3.3 Criteria for Subject Exclusion

Subjects with the following conditions will be excluded from the study:

3.3.1 PSA ≥ 100 ng/mL.

3.3.2 Prostate volume > 100 cc.

3.3.3 Pathologically positive lymph nodes or nodes > 1.0 cm on imaging. Note: nodes > 1.0 cm but biopsy negative are allowed.

3.3.4 Evidence of M1 metastatic disease.

3.3.5 Prior invasive malignancy except for non-melanoma skin cancer within 5 years of enrollment. Subjects must be disease-free for > 5 years.

3.3.6 Prior radical prostatectomy, cryosurgery for prostate cancer, or bilateral orchiectomy for any reason.

3.3.7 If the subject had prior androgen deprivation therapy (ADT), the subject exhibited biochemical failure while on ADT.

3.3.8 Prior systemic chemotherapy for the study cancer. Note that prior chemotherapy for a different cancer is allowed; however, subjects must be > 2 years post-completion of chemotherapy at time of registration. Subjects on Proscar therapy must stop to be eligible.

3.3.9 Major surgery planned within 3 months of registration.

3.3.10 Severe, active co-morbidity defined as:

3.3.10.1 New York Heart Association Class II or greater congestive heart failure or active ventricular arrhythmia requiring medication.

3.3.10.2 Chronic obstructive pulmonary disease (COPD) exacerbation or other respiratory illness requiring hospitalization within last 3 months or precluding study therapy at the time of registration.

3.3.10.3 Acute infection. Acute infection is defined by any viral, bacterial, or fungal infection that requires specific therapy within 72 hours of initiation of the study therapy.

3.3.11 Previous history of liver disease including hepatitis.

3.3.12 Immunosuppressive therapy including systemic corticosteroids. Use of inhaled and topical corticosteroids is permitted.

3.3.13 Impaired immunity or susceptibility to serious viral infections.

3.3.14 Allergy to any product used in the protocol. If the subject has an allergy to Ciproflaxin, another antibiotic can be substituted at the discretion of the treating physician.

3.3.15 Serious medical or psychiatric illness or concomitant medication, which, in the judgment of the principal investigator, might interfere with the subject’s ability to respond to or tolerate the treatment or complete the trial.

4.0 REGISTRATION PROCEDURES

4.1 Registration and Enrollment

4.1.1 Subjects can be registered to the trial only after the following conditions have been met:

4.1.1.1 All inclusion/exclusion criteria of the study are met.

4.1.1.2 The Informed Consent has been signed and witnessed.

The following registration procedures will be used:

4.1.2 Once an eligible subject who meets the inclusion/exclusion criteria of the study has signed the Informed Consent, and eligibility case report form (CRF) will be completed by the clinical research staff. The Data Collection Center (DCC) in the Department of Health Services (PHS) will be notified with tentative treatment start date through the eligibility CRF.

4.1.3 The subject is considered to be enrolled into the trial if/when he receives the adenovirus injection.

4.1.4 A subject schedule will be generated by the DCC for clinical assessments/visits and medications when the subject's enrollment information is entered by the clinical staff.

4.2 Study Drug Accountability

Dr. Freytag will be responsible for the preparation, allocation and disposal of the Ad5-yCD/*mut*TK_{SR39}*rep*-hIL12 adenovirus. He will also be responsible for maintaining the study drug inventory and log. The study drug inventory will be reviewed by the DCC on a regular basis for accountability.

5.0 STUDY THERAPY AND PROTOCOL

5.1 Treatment Plan

5.1.1 Pretreatment Evaluations

5.1.1.1 History and physical examination including digital rectal exam (DRE).

5.1.1.2 Hematology- CBC/DP.

5.1.1.3 Blood chemistries- glucose, Na, K, Cl, Ca, Mg, P, CO_2, BUN, creatinine, AST/SGOT, ALT/SGPT, ALKP, bilirubin, serum protein, albumin, globulin, GFR, CPK, LDH, GGT, testosterone.

5.1.1.4 Diagnostic serum PSA.

5.1.1.5 Urinalysis, urine culture, and sensitivity.

5.1.1.6 Chest radiographs (PA and lateral).

5.1.1.7 Diagnostic imaging- bone scan and CT scan of the abdomen and pelvis within 90 days of registration.

5.1.1.8 Biopsy/pathology reviews- a TRUS-guided needle biopsy of the prostate (≥ 6 core) must be performed within 180 days of registration to confirm the presence of adenocarcinoma and to determine prostate volume. Biopsy cores should be kept separate, coded and submitted to Department of Pathology for analysis.

5.1.1.9 Molecular studies- presence of Ad5-yCD/*mut*TK_{SR39}*rep*-hIL12 in blood by polymerase chain reaction (PCR). This is a baseline control for the post-treatment PCR assays and does not have to be completed before the subject can be enrolled.

5.1.1.10 Immunological studies- serum IL-12 and IFN-γ will be measured by enzyme-linked immunosorbent assay (ELISA). NK cell cytolytic activity will be measured by ^{51}Cr release or non-radioactive cytotoxicity assay. These are baseline controls for the post-treatment evaluations and do not have to be completed before the subject can be enrolled.

5.1.1.11 EPIC and EQ-5D instruments.

5.1.1.12 The subject will be required to meet with a urologic surgeon to discuss other treatments options such as salvage prostatectomy. This meeting must be documented in the patient’s records.

5.1.2 Oncolytic Adenovirus-Mediated Gene Therapy

5.1.2.1 Description of Ad5-yCD/*mut*TK_{SR39}*rep*-hIL12 Adenovirus

Ad5-yCD/*mut*TK_{SR39}*rep*-hIL12 is a replication-competent, type 5 adenovirus (Ad5) containing a yeast CD (yCD)/mutant SR39 HSV-1 TK (HSV-1 TK_{SR39}) fusion gene in the E1 region and human IL-12 coding sequences in the E3 region (57). Expression of the yCD/HSV-1 TK_{SR39} fusion gene renders tumor cells sensitive to 5-FC and vGCV. Expression of IL-12 stimulates the innate and adaptive arms of immunity, inhibits angiogenesis, and mitigates immune suppression in the tumor milieu.

5.1.2.2 Supply of Ad5-yCD/*mut*TK_{SR39}*rep*-hIL12 Adenovirus

Clinical-grade Ad5-yCD/*mut*TK_{SR39}*rep*-hIL12 adenovirus was manufactured at the Baylor College of Medicine Vector Production Facility (Houston, TX) using GMP procedures. The adenovirus was supplied as a sterile, frozen liquid in vials containing 1.1 mL at a concentration of 1×10^{12} viral particles (vp) per milliliter (mL).

5.1.2.3 Storage of Ad5-yCD/*mut*TK$_{SR39}$*rep*-hIL12 Adenovirus

The Ad5-yCD/*mut*TK$_{SR39}$*rep*-hIL12 adenovirus is stored in a locked ultralow (-80°C) freezer located in a room with restricted access. An inventory log is maintained by Dr. Freytag.

Stability testing of the Ad5-yCD/*mut*TK$_{SR39}$*rep*-hIL12 adenovirus will be performed on a semi-annual basis. This will involve: 1) determination of titer, 2) genetic stability as determined by restriction digest analysis of adenoviral DNA, 3) quantification of the adenovirus cytopathic effect following infection of human prostate adenocarcinoma cells, 4) ability of the adenovirus to confer 5-FC and GCV sensitivity onto infected prostate adenocarcinoma cells, and 5) IL-12 expression following infection of human prostate adenocarcinoma cells.

5.1.2.4 Handling and Dilution of Ad5-yCD/*mut*TK$_{SR39}$*rep*-hIL12 Adenovirus

Human adenoviruses are Risk Group 2 (RG2) agents and should be handled using Biosafety Level 2 (BL2) procedures (67).

Thirty minutes prior to each injection, a new vial of Ad5-yCD/*mut*TK$_{SR39}$*rep*-hIL12 adenovirus will be removed from the storage freezer, thawed rapidly and placed on ice. The adenovirus will be diluted with sterile saline to the prescribed concentration. The diluted adenovirus will be loaded into 1 mL disposable syringes and placed on ice in a Styrofoam container. The container will be sealed with tape and transported to the treatment room.

The manufacturer, lot number and expiration date of the sterile saline used to dilute the adenovirus will be recorded.

5.1.2.5 Ad5-yCD/*mut*TK$_{SR39}$*rep*-hIL12 Adenovirus Dose

The treatment schema is depicted in **Figure 5**.

Dosing of the Ad5-yCD/*mut*TK$_{SR39}$*rep*-hIL12 adenovirus is shown in **Table 5**. The starting dose level will be 1 x 10^{10} vp. The interval between dosing of the first two subjects in the first cohort should be ≥ 2 weeks.

All subjects in each cohort will be evaluated for ≥ 30 days before proceeding to the next cohort. If one of three subjects experiences a dose-limiting toxicity (DLT) (defined in Section **6.3.2.3**), three additional subjects will be enrolled at that dose level. If only one of six subjects experiences a DLT at a given dose level, the dose will escalate to the next cohort of three subjects. If two subjects experience a DLT at a given dose level, the maximum tolerated dose (MTD) is exceeded. Three

additional subjects will be enrolled at the previous dose level if there were only three subjects at that dose. The adenovirus dose will be escalated to a maximum of 1 x 10^{12} vp.

Table 5. Ad5-yCD/*mut*TK$_{SR39}$*rep*-hIL12 Dosing

Cohort	# Subjects	Adenovirus Dose (vp)
1	3 to 6	1 x 10^{10}
2	3 to 6	3 x 10^{10}
3	3 to 6	1 x 10^{11}
4	3 to 6	3 x 10^{11}
5	3 to 6	1 x 10^{12}

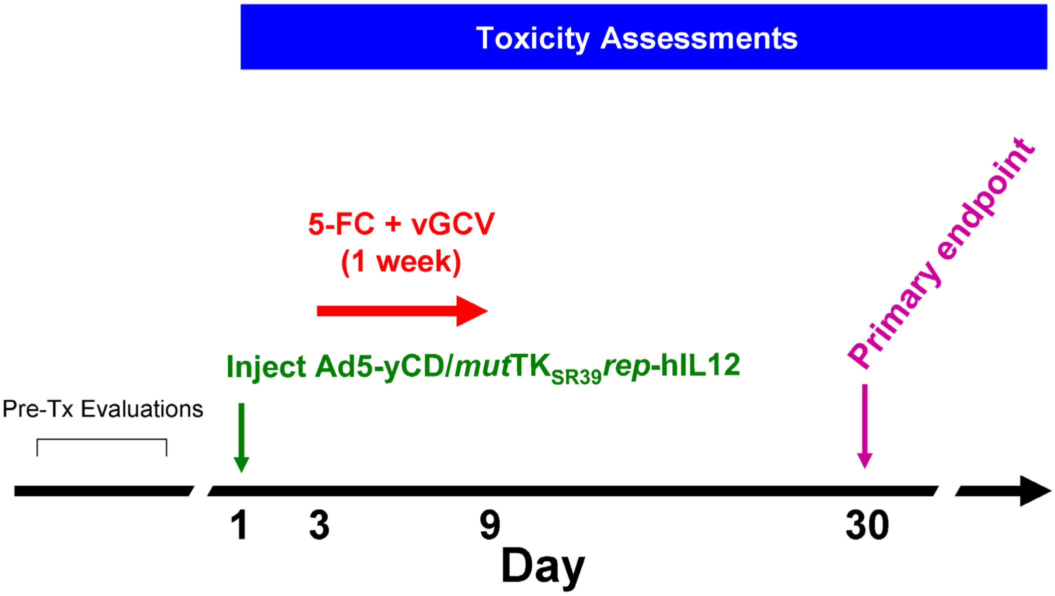

Figure 5. Treatment Schema. Subjects will undergo a series of pre-treatment evaluations and must meet all of the eligibility criteria to be enrolled in the study. Subjects will receive a single intraprostatic injection of the Ad5-yCD/*mut*TK$_{SR39}$*rep*-hIL12 adenovirus at one of five dose levels (see Table 5) on day 1. Two days later (day 3), subjects will receive a one week (7 day) course of 5-FC (150 mg/kg/day) + vGCV (1,800 mg/day) prodrug therapy. Toxicity assessments will occur twice a week during the first two weeks and then at every scheduled follow-up visit. The primary endpoint is toxicity through day 30.

5.1.2.6 Injection Volume

The total injection volume will be 3.0 - 4.0 mL depending on the size of the prostate.

5.1.2.7 Intraprostatic Injection Procedure

Subjects will be seen as outpatients the day of the adenovirus injection. The injection procedure will be performed in a private treatment room with restricted access. Biosafety Level 2 procedures will be followed throughout.

Adenovirus injections will be performed transrectally under transrectal ultrasound guidance (TRUS) typically using 20 gauge needles. The subject will be placed in the left lateral decubitus position with the legs flexed and the knees brought up toward the chest. The buttocks are placed over the edge of the examining table to allow for the appropriate manipulation of the ultrasound probe. Prior to the adenovirus injection, the subject will receive a local injection of 1% lidocaine into the area surrounding the prostatic capsule. Two needle passes (one medial, one lateral) will be made in the left and right sides of the prostate starting at the base and proceeding toward the apex until all of the adenovirus is delivered.

Used syringes and needles will be disposed of properly. After the procedure, the treatment room will be cleaned with an approved disinfectant.

5.1.2.8 Toxicity

Injection procedure- Possible side effects of the injection procedure include infection, gross hematuria and urinary retention. Infection occurs in less than 1% of cases. Antibiotic therapy will be given as prophylaxis prior to and after the adenovirus injection. Gross hematuria following prostate biopsy occurs in less than 1% of cases and is usually self-limited. Injection of the adenovirus into the prostate may cause local swelling that could lead to acute urinary retention. A Foley catheter will be placed, if needed.

Ad5-yCD/*mut*TK_{SR39}*rep*-hIL12 adenovirus- Based on the results of five phase 1/2 trials with similar oncolytic adenoviruses lacking IL-12 (36-44), likely adverse events attributable to the oncolytic adenovirus include transient flu-like symptoms (37 of 79 subjects or 47%) and transaminitis (32 of 79 subjects or 41%). The vast majority (> 94%) of these events were mild (grade 1) to moderate (grade 2). The flu-like symptoms typically resolve within a few days and can be alleviated with analgesics. The transaminitis is self-limiting and typically resolves within 2 weeks.

The toxicities associated with IL-12 administration are well-established (59, 62, 63), some of which overlap those associated with the adenovirus and prodrug therapy. It is possible/likely that IL-12 will exacerbate flu-like symptoms (e.g., chills/rigor, fever, malaise), hepatic toxicity (e.g., transaminitis, ALKP, bilirubin), and hematological events (e.g., anemia, leukopenia, lymphopenia, neutropenia, thrombocytopenia). Other adverse events possibly/probably related to IL-12 administration include: ascites, confusion, capillary leak syndrome, cytokine release syndrome, dehydration, delirium, diarrhea, dizziness, dyspnea, edema, headache, hypotension, nausea, pleural effusion,

tachycardia, and vomiting.

5.1.2.9 Dose Modification

Subjects will receive only a single adenovirus injection. No dose modifications are necessary.

5.1.3 Prodrug Therapy

5.1.3.1 Description

5-fluorocytosine (5-FC, Ancobon®) is approved for the treatment of serious infections caused by susceptible strains of Candida and Cryptococcus. Valganciclovir (vGCV, Valcyte®) is approved for the treatment of CMV retinitis in people with AIDS.

5.1.3.2 Supply of 5-FC and vGCV Prodrugs

Both 5-FC (Ancobon®) and vGCV (Valcyte®) are commercially available. 5-FC (Ancobon®) is manufactured by Valeant Pharmaceuticals and is available as 250 and 500 mg capsules for oral administration. vGCV (Valcyte®) is manufactured by Hoffman La-Roche and is available as 450 mg tablets for oral administration.

5.1.3.3 Storage of 5-FC and vGCV Prodrugs

5-FC (Ancobon®) and vGCV (Valcyte®) should be stored as directed by the supplier.

5.1.3.4 Duration of Prodrug Therapy

All subjects will receive a one week (7 day) course of 5-FC (Ancobon®) + vGCV (Valcyte®) prodrug therapy beginning two days after the adenovirus injection (**Figure 5**).

5.1.3.5 5-Fluorocytosine (5-FC, Ancobon®) Dose

The 5-FC (Ancobon®) dosage will be 150 mg/kg per day given in four equally divided doses. Due to 5-FC (Ancobon®) only being available in 250 and 500 mg capsules, the dose may be rounded up or down to the nearest possible amount. The capsules will be taken a few at a time over a 15 minute period.

5.1.3.6 Valganciclovir (vGCV, Valcyte®) Dose

The vGCV (Valcyte®) dosage will be 1,800 mg/day given twice daily (2 x 450 mg tablets per dose) with food.

5.1.3.7 Toxicity

The most common side effects associated with 5-FC (Ancobon®) + vGCV (Valcyte®) prodrug therapy include neutropenia and thrombocytopenia. In five previous phase 1/2 trials representing 79 subjects (36-44), 19 of 79 (24%) subjects exhibited grade 1 - 3 neutropenia and 26 of 79 (33%) subjects exhibited grade 1/2 thrombocytopenia. The prodrugs may also contribute to anemia (32 of 79 subjects or 41%) and possibly lymphopenia (69 of 79 subjects or 87%). Except for lymphopenia, the vast majority of these events are mild (grade 1) to moderate (grade 2). Sixty-four percent (64%) of the events of lymphopenia were moderate (grade 2) and 25% were severe (grade 3). All hematologic events are transient and self-limiting, and typically resolve within two (anemia, neutropenia, thrombocytopenia) to six (lymphopenia) weeks.

Other possible side effects associated with 5-FC (Ancobon®) + vGCV (Valcyte®) prodrug therapy include nausea, vomiting, diarrhea and headache.

5.1.3.8 Dose Modifications

5-FC and vGCV doses will be modified based on the guidelines below.

5.1.3.8.1 Cytopenia

5.1.3.8.1.1 Absolute neutrophil count < 1,000/µL or platelet count < 100,000/µL- reduce 5-FC and vGCV doses to 75%.

5.1.3.8.1.2 Absolute neutrophil count < 500/µL or platelet count < 50,000/µL- discontinue 5-FC and vGCV, and resume the drugs at 50% of the original dose when the neutrophil count is > 500/µL and platelets > 50,000/µL.

5.1.3.8.2 Creatinine Clearance

If creatinine clearance (CrCl) is < 50 mL/min/m^2, follow the guidelines in **Table 6** and check the creatinine every other day (except weekends). Creatinine clearance should be calculated based on the most recent creatinine level as follows:

$$CrCl = \frac{(140 - \text{age [years]}) \times (\text{body weight [kg]})}{(72) \times (\text{serum creatinine [mg/dL]})}$$

**Table 6. Ancobon and Valcyte Dose Modifications**		
**Creatinine Clearance**	**Ancobon**	**Valcyte**
≥ 50	No modifications	No modifications
≥ 40 and < 50	Reduce to 75 mg/kg/day	Reduce to 450 mg b.i.d.
≥ 25 and < 40	Reduce to 37.5 mg/kg/day	Reduce to 450 mg q.d.
< 25	Suspend	Suspend

In all cases, if creatinine clearance improves, Ancobon and Valcyte doses can be increased (resumed) once according to guidelines in **Table 6**. If creatinine clearance drops a second time, Ancobon and Valcyte dosage should be reduced accordingly, but may not be increased if creatinine clearance improves. In all cases, the subject should take no more than a maximum of 7 days of prodrug medication (regardless of the dose received). If prodrugs are suspended continuously for > 2 weeks, the subject should not receive any further prodrugs.

5.1.4 Other Medications

5.1.4.1 Antibiotics

To reduce the risk of infection, subjects will be given ciprofloxacin (500 mg, p.o., b.i.d.) the evening before, and the morning of, the adenovirus injection, continuing the antibiotic twice daily for a total of 3 days. They will also receive gentamicin (120 mg, I.M.) just prior to the adenovirus injection. If the subject is allergic to ciprofloxacin, another antibiotic can be substituted at the discretion of the treating physician.

6.0 SUBJECT ASSESSMENTS

Subject assessments are summarized in **Table 7**.

Table 7. Subject Assessments

Parameters	Pre-Study Therapy	First 2 Weeks	Follow Up
History, Physical Examination, DRE	X[a]		X[i]
EPIC and EQ-5D Instruments	X[a]		X[j]
Bone Scan	X[a]		X[k]
CT Scan	X[a]		X[k]
On-site Physician Assessment	X[b]	X[e]	X[i]
Hematology (CBC/DP)	X[b]	X[f]	X[i]
Blood Chemistries	X[b]	X[f]	X[i]
Urinalysis	X[b]		
Diagnostic Serum PSA	X[b]	X[e]	X[i]
Toxicity Assessment	X[b]	X[f]	X[i]
Molecular Studies	X[b]	X[g]	X[f]
IL-12 and IFN-γ ELISA	X[b]	X[g]	X[f]
NK Cytolytic Assay	X[b]	X[h]	
Chest Radiographs	X[c]		
TRUS-Guided Needle Biopsy	X[d]		
Pathology Assessment	X[d]		

[a]Within 90 days of registration. [b]Within 30 days of registration. [c]Within 90 days of registration if the subject has history of pulmonary disease; otherwise, within 180 days. [d]Within 180 days of registration. [e]Once a week for the first two weeks. [f]Twice a week for the first 2 weeks. [g]At every blood draw until not detected in two consecutive measurements. [h]At every blood draw during the first two weeks. [i]At every scheduled follow-up visit at 1, 3, 6, 9, 12, 18, 24 months and then annually thereafter. [j]At 6, 12, and 24 months. [k]In subjects who experience bone pain or at PSA relapse and then annually thereafter.

6.1 Assessments During First Two Weeks After Adenovirus Injection

The following assessments will be performed:

6.1.1 An on-site physician assessment will be performed once a week.

6.1.2 CBC/DP and blood chemistries will be measured twice a week, or as clinically indicated.

6.1.3 Diagnostic serum PSA.

6.1.4 Ad5-yCD/*mut*TK$_{SR39}$*rep*-hIL12 viral DNA in blood will be measured at every scheduled blood draw until not detected in two consecutive measurements.

6.1.5 Serum IL-12 and IFN-γ will be measured at every scheduled blood draw until not detected in two consecutive measurements.

6.1.6 NK cytolytic activity will be measured at every scheduled blood draw during the first two weeks after the adenovirus injection.

6.1.7 Toxicity assessments will be performed twice a week using protocol-specific Case Report Forms (CRFs). Toxicity assessments will be based on physical examinations, diagnostic evaluations, and laboratory tests.

6.2 Assessments Following the Study Therapy

Follow-up visits will occur at 1, 3, 6, 9, 12, 18 and 24 months after the adenovirus injection and then at least annually thereafter. Evaluations will include:

6.2.1 History and physical examination including DRE.

6.2.2 CBC/DP and blood chemistries.

6.2.3 Diagnostic serum PSA.

6.2.4 EPIC and EQ-5D instruments at 6, 12, and 24 months.

6.2.5 Ad5-yCD/*mut*TK_{SR39}*rep*-hIL12 viral DNA in blood will be measured at every scheduled blood draw until not detected in two consecutive measurements.

6.2.6 Serum IL-12 and IFN-γ will be measured at every scheduled blood draw until not detected in two consecutive measurements.

6.2.7 A bone scan will be performed on any subject with complaints of bone pain that cannot be attributed to any intercurrent disease. Discretionary plain films and/or MRI may be needed to evaluate lesions seen on bone scan to confirm the diagnosis of metastatic disease.

6.2.8 In subjects with biochemical failure, a bone scan and CT scan of the abdomen and pelvis should be performed at that time and then annually and/or as clinically indicated.

6.2.9 Toxicity assessments will be performed at every scheduled follow-up visit using CRFs specifically designed for this trial. Toxicity assessments will be based on physical examinations, diagnostic evaluations, and laboratory test results.

6.2.10 It is understood that the performance of an individual study or test as specified in this protocol is subject to factors such as subject compliance, schedule difficulties, equipment malfunction, or the judgment of the principal investigator or patient care physicians, and that a test may not be done in an individual instance without violating the protocol. However, any systematic modification of the original protocol in this regard, whether related to subject safety or not, will be submitted to the IRB for approval.

6.3. Toxicity Assessments

6.3.1 Adverse Event Grading Criteria

Adverse events will be graded according to NCI's Common Terminology Criteria for Adverse Events (CTCAE) v4.03 (68). Adverse events will be documented using CRFs specifically designed for this trial.

6.3.2 Definitions

6.3.2.1 Definition of Adverse Event (AE)

An adverse event is defined as any undesirable experience associated with the use of a medical product (69). Adverse events will be categorized as depicted in **Table 8** (70).

Table 8. Categorization of Adverse Events

Relationship	Attribution	Description
**Unrelated** to investigational agent/intervention	Unrelated	The AE *is clearly **NOT** related* to agent/intervention
	Unlikely	The AE *is **doubtfully related*** to agent/intervention
**Related** to investigational agent/intervention	Possible	The AE ***may be related*** to agent/intervention
	Probably	The AE ***is likely related*** to agent/intervention
	Definite	The AE ***is clearly related*** to agent/intervention

6.3.2.2 Definition of Serious Adverse Event (SAE)

A SAE is defined as any undesirable experience associated with the use of a medical product that results in any of the following outcomes:

6.3.2.2.1 Death.

6.3.2.2.2 A life-threatening experience.

6.3.2.2.3 Hospitalization or prolongation of existing hospitalization.

6.3.2.2.4 Disability or permanent damage.

6.3.2.2.5 A congenital anomaly/birth defect.

6.3.2.2.6 Required intervention to prevent permanent impairment or damage.

6.3.2.2.7 Other serious events that may jeopardize the subject and may require medical or surgical intervention (treatment) to prevent one of the other outcomes.

6.3.2.3 Definition of Dose-Limiting Toxicity (DLT)

Dose-limiting toxicity is defined as any of the following adverse events considered possibly/probably/definitely related to the study treatment.

6.3.2.3.1 ≥ Grade 2 allergic reaction or generalized urticaria.

6.3.2.3.2 ≥ Grade 2 cardiovascular or neurologic toxicity.

6.3.2.3.3 ≥ Grade 3 hematologic or non-hematologic toxicity of any duration except for:

6.3.2.3.3.1 Grade 3 lymphopenia with recovery within 2 weeks.

6.3.2.3.3.2 Grade 3 anemia, leukopenia, or neutropenia that is reversible within 72 hours.

6.3.2.3.3.3 Grade 3/4 fever or flu-like symptoms that are reversible within 72 hours.

6.3.2.3.3.4 Grade 3 SGOT/SGPT that is reversible within 72 hours.

6.3.2.3.3.5 Grade 3 hyperglycemia that can be explained by patient's baseline co-morbidity (i.e., diabetes).

6.3.2.3.3.6 Grade 3 hypermagnesemia that is reversible within 72 hours.

6.3.2.3.3.7 Grade 3 hypophosphatemia that is reversible within 72 hours.

6.3.2.4 Definition of Maximum Tolerated Dose (MTD)

MTD is defined as the highest Ad5-yCD/*mut*TK_{SR39}*rep*-hIL12 dose at which < 33% (< 1 of 3 or < 2 of 6) of any cohort develops DLT.

If 1 of 3 subjects in a cohort develops DLT, the cohort will be expanded to 6 subjects. If ≥ 2 subjects out of 6 in a cohort develop DLT, MTD will have been exceeded at that dose. If there is a lower dose and if the cohort at that dose has not already been expanded to 6, the cohort will be expanded to 6 at the lower dose. If ≥ 2 subjects develop DLT, MTD will have been

exceeded and the trial will stop.

6.3.3 Adverse Event Reporting Guidelines

Guidelines for adverse event reporting are depicted in **Table 9**. Adverse events that require reporting will be reported using the Adverse Event Reporting CRF specifically designed for this study.

Table 9. Adverse Event Reporting Requirements

Not Serious	Serious AND *unrelated* to the agent/intervention	Serious AND *possibly, probably or definitely related* to the agent/intervention
Reporting is NOT required.	Reporting is NOT required	Report by phone/FAX/email to IRB within 24 hours of discovery of event. Written report to IRB, IBC, OBA and FDA within 10 calendar days. This includes deaths within 30 days of adenovirus injection.

6.4 Subject Removal and Study Stopping Rules

6.4.1 Subject Removal Rules

Every attempt will be made to obtain follow-up on subjects who have received the study medication.

6.4.1.1 The subject may withdraw from the study upon request of the participating subject or person with the power of attorney.

6.4.1.2 Noncompliance.

6.4.1.3 Intercurrent medical or psychiatric problem or institution of a concomitant medication that interferes with compliance with the protocol or places the subject at increased risk.

6.4.1.4 Dose-limiting toxicity (DLT).

6.4.1.5 Investigator's discretion, to the extent that subsequent clinical study requirements place the subject at unacceptable risks.

6.4.2 Study Stopping Rules

6.4.2.1 DLT in the first dose cohort.

6.4.2.2 If > 33% of enrolled subjects develop ≥ grade 3 hematuria or urinary retention, the study will be stopped and the injection procedure re-evaluated.

6.4.2.3 Transmission of the Ad5-yCD/*mut*TK_{SR39}*rep*-hIL12 adenovirus to any subject contacts, including health care personnel.

6.4.2.4 Lack of availability of the Ad5-yCD/*mut*TK_{SR39}*rep*-hIL12

adenovirus.

6.4.2.5 Insufficient accrual.

6.5 Measurement of Treatment Effect

Treatment effect will be based on post-treatment digital rectal examinations (DRE), PSA evaluations, bone and CT/MRI scans. DRE and PSA will be assessed at every scheduled follow-up visit. Bone and CT/MRI scans will be performed as clinically indicated.

6.6 Definition of Response Parameters

6.6.1 Freedom from Biochemical (PSA) Failure- The time to PSA failure will be measured from the date of the adenovirus injection to the date of a rise of ≥ 2 ng/mL above the nadir PSA (71). Nadir PSA is defined as the lowest PSA value after the adenovirus injection and before the call date of PSA failure. The time of failure will be the date of the first PSA that is ≥ 2 ng/mL above the post-treatment nadir.

6.6.2 Freedom from Clinical Failure- The time to clinical failure (local and/or regional/distant) will be measured from the date of the adenovirus injection to the date that clinical failure is first documented.

Local clinical failure is defined as having any ONE of the following:

6.6.2.1 Local progression, as measured by a ≥ 25% increase in the product of the two largest perpendicular diameters of the prostate tumor at any time.

6.6.2.2 Failure of regression of palpable tumor by 2 years.

6.6.2.3 Redevelopment of a palpable abnormality after complete disappearance of previous abnormalities.

Note: In all above cases, unless clinically contraindicated, needle biopsy verification is required to document the presence of local tumor.

Regional/distant clinical failure is defined as having any ONE of the following:

6.6.2.4 Radiographic (CT or MRI) evidence of new or increased pelvic lymphadenopathy (measuring >1.5 cm on short axis) and/or histological confirmation of tumor.

6.6.2.5 The development of a new area of isotope uptake on bone scan relative to baseline that cannot be attributed to trauma and is clinically consistent with metastatic disease. Any questionable findings must be confirmed by other imaging modalities (e.g., MRI) and/or biopsy.

6.6.2.6 The development of a clinical and/or radiographic finding

consistent with metastatic disease (e.g., a pulmonary metastasis).

6.6.3 Disease-Specific Survival- The time to disease-specific death will be measured from the date of the adenovirus injection to the date of death due to prostate cancer. Causes of death may require review by the study PI or designee. Death due to prostate cancer will be defined as:

6.6.3.1 Primary cause of death certified as due to prostate cancer.

6.6.3.2 Death in association with any ONE of the following conditions:

6.6.3.2.1 Further clinical tumor progression occurring after initiation of additional salvage therapy such as ADT.

6.6.3.2.2 A rise in serum PSA that exceeds 1.0 ng/mL on at least two consecutive occasions during or after salvage ADT.

6.6.3.2.3 Disease progression in the absence of any prostate cancer therapy.

6.6.3.2.4 Death from a complication of prostate cancer therapy, irrespective of disease status.

6.6.4 Survival- The survival time will be measured from the date of the adenovirus injection to the date of death. All subjects will be followed for survival. Every effort should be made to document the cause of death. Post-mortem examination will be carried out when feasible and a copy of the final autopsy report should be sent to the DCC.

7.0 DATA COLLECTION

7.1 Data Collection and Management

The Data Coordinating Center (DCC) is headed by Jingfang Cheng, RN, and is located in the Department of Radiation Oncology, Henry Ford Health System (HFHS). Ms. Chang has over 10 years experience in managing clinical trial databases. The DCC will use a Microsoft Access database for data collection using a set of Standard Operating Procedure (SOPs) developed by Ms. Cheng and the study coordinator.

7.2 Standard Operating Procedures

Standard Operating Procedures (SOPs) have been developed by Ms. Cheng and the study coordinator to be compliant with FDA regulations and implemented at the DCC for FDA regulated clinical trials. SOPs were developed using the FDA Good Clinical Practice Guidelines.

7.3 Case Report Forms

Case Report Forms (CRFs) have been developed specifically for this trial. The

database will be created to match with CRFs so that the data structure provides fast, efficient, and accurate data processing.

7.4 Database and Data Management

The database for this study will be implemented using a Microsoft Access database housed on a secure departmental drive. Access is a relational database with security options that limit the ability to access the data to specified people. The database structure will be set up according to the CRFs. Specific queries can be run to check for data discrepancies and missing fields. An independent monitor will conduct audits of the data for completion and accuracy no less than one time per year.

7.5 Plan for the Site Initialization

Subjects will be enrolled at the Henry Ford Health System only. After receiving all required federal (FDA, OBA) and local (IRB, IBC) approvals, Drs. Stricker, Freytag and Cheng will conduct the site initiation. They will meet with the study nurse and clinical coordinator to discuss the following: study drug, study protocol, subject enrollment, drug accountability, CRFs and instructions for completion of CRF and CRF submission, and adverse event (AE) reporting. A follow-up meeting(s) will be conducted, if one meeting is not sufficient. During subject enrollment and follow-up, Drs. Stricker and Freytag, and Ms. Cheng, will be available to answer questions regarding administrative issues, enrollment, CRF data collection and quality assurance. The key investigators will meet on a regular basis to go over issues encountered during the study.

7.6 Subject Registration

Subject registration is described in **Section 4.0**. After receiving the registration information, the DCC will generate the subject's schedule for follow-up.

7.7 CRF Data Collection and Data Management

CRF data is collected based on the sources of truth (e.g., medical records). Data sources will include subject interviews (subject history, adverse events), physical examination, histology/pathology reports, all laboratory data, and medical records. Subjects will be identified by study subject number and initials (first, middle, last); if there is no middle initial, a hyphen will be used (first-last). Last names with apostrophes will be identified by the first letter of the last name. Completed CRFs will be entered into the study database by study staff on a regular basis.

Data entry is summarized in **Table 10**.

Table 10. Summary of Data Submission	
**Case Report Form**	**Due**
Eligibility Checklist	At registration
Registration Form	Within 2 weeks of registration
Pre-Treatment Conditions	Within 2 weeks of registration
Concomitant Medications	Within 2 weeks of registration
Adenovirus Injection Summary	Within 2 weeks of adenovirus injection
Prodrug Therapy	Within 2 weeks of completion of prodrug therapy regimen
Blood Labs	Within 2 weeks of blood draw
Toxicity Assessment	Within 2 weeks of toxicity assessment
SAE That Require Reporting	-24 h by phone/FAX/email<br>-Written report within 10 calendar days
Adenoviral DNA in Blood	Within 90 days of adenovirus injection
On-site Physician Assessment	Within 2 weeks of assessment
Discontinuation of Study Therapy	Within 2 weeks of discontinuation
Death Report	If ≤ 30 days from adenovirus injection:<br>-24 h by phone/FAX/email<br>-Written report within 10 calendar days<br>If > 30 days from adenovirus injection:<br>-Within 2 weeks of learning of event
Long-Term Follow-Up	Within 2 weeks of assessment
EPIC and EQ5D	Within 2 weeks of assessment

7.8 Quality Assurance Plan to Ensure Data Quality

Data quality assurances (QA) are applied both at the DCC and clinical sites. Features of the QA plan at the DCC include:

7.8.1 Initial review of forms

7.8.2 Check for possible missing forms

7.8.3 CRF data field checks for missing, miscodes and outliers

7.8.4 Code for tracking person who completed forms

7.8.5 Data consistency checks (within or across the forms)

7.8.6 Generation of queries and an electronic audit trail

All QA queries will be documented by the data manager with respect to:

7.8.7 Query generation date

7.8.8 Date query sent to clinical site for resolution

7.8.9 Identification information for the form

7.8.10 Old (or original) value

7.8.11 Error message(s)
7.8.12 Code of person who completed the form

7.8.13 New value (may not represent a change)

7.8.14 Reason for new value

7.8.15 ID of site staff member responsible for new value

7.8.16 Date change made to database

Report forms are produced from a query table and sent to the PI for resolution. Data are processed as they are collected to ensure the smooth and systematic flow of activities, and to avoid backlogs and uneven workloads.

7.9 Confidentiality

Throughout the data collection period, the data of individual subjects will be kept confidential by DCC staff. The clinical site only holds names or other personal identifiers for conducting the subject's follow-up. Subjects' names and other personal identifiers can not appear on any data collection form, CRFs and analysis reports. The DCC must keep these strictly confidential among staff.

8.0 STATISTICAL CONSIDERATIONS

This phase 1 trial is designed to determine the toxicity of oncolytic adenovirus-mediated cytotoxic and IL-12 gene therapy in locally recurrent prostate cancer. Endpoints are classified as primary, secondary and exploratory.

8.1 Endpoints, Hypotheses and Analyses

8.1.1 Primary Endpoint

8.1.1.1 Hypothesis- Oncolytic adenovirus-mediated cytotoxic and IL-12 gene therapy is associated with acceptable toxicity.

Adverse events will be graded according to NCI's Common Terminology Criteria for Adverse Events (CTCAE) v4.03 (68). Adverse events will collected through day 30 (day 1 being the day of the adenovirus injection).

8.1.2 Secondary Endpoints

8.1.2.1 Hypothesis 1- Oncolytic adenovirus-mediated cytotoxic and IL-12 gene therapy has an effect on serum PSA levels.

Serum PSA will be assessed according to Table 7 and compared to the subject's baseline value.

8.1.2.2 Hypothesis 2- Oncolytic adenovirus-mediated cytotoxic and IL-12 gene therapy has an effect on PSADT.

Serum PSA will be assessed according to Table 7. PSADT will be determined by logarithm regression analysis as described previously (38). PSADT prior to the study therapy will be calculated using a minimum of three PSA values including the last PSA prior to initiation of the study therapy. Subjects who do not have at least three pre-treatment PSA values will be excluded from the analysis. PSADT after the study treatment will be calculated from the nadir and will use all PSA values prior to the implementation of additional salvage therapy such as ADT.

PSADT will be calculated assuming first-order kinetics using the following equation:

$$Ln\ (\mathrm{PSA}) = \beta_o + \beta_1 t$$

where β_o is the point where the regression line crosses the ordinate at t = 0 and β_1 is the number of units that Ln (PSA) changes for every one-unit change in time (i.e., the slope). PSADT is defined as $Ln\ (2)\ /\ \beta_1$. Data are evaluated for normality the ranked data are considered if data are not normal.

8.1.2.3 Hypothesis 3- Oncolytic adenovirus-mediated cytotoxic and IL-12 gene therapy has no significant effect on quality of life.

We will use two instruments to measure QOL: EPIC and EQ-5D. QOL instruments will be collected on all subjects at baseline, 6, 12 and 24 months after the adenovirus injection.

EPIC questionnaires will be completed by the subject. The questionnaires will be summarized into three endpoints of function and bother in urinary, bowel and sexual domains with a score of 0 to 100. EQ-5D will have a self-report score of 1 to 3 in five domains (mobility, self-care, usual activity, pain/discomfort and anxiety/depression), and an overall health score 10 to 100. If a subject death is related to prostate cancer or the study treatment, the worst score will be given and the subject will be included in the analysis.

Data will be evaluated for normality, data transformation or nonparametric approach will be considered if data are not normal.

For each QOL domain variable, analysis of variance and covariance (ANCOVA) will be used. Analysis will include the baseline covariates of pre-treatment domain score, covariate of time (of assessment post-treatment) and the independent

treatment variable. The analysis will start testing for treatment by time interaction, followed by testing the treatment effect at each time point if the interaction is detected at critical value 0.10, or testing the overall treatment effect or time effect at critical value of 0.05 if otherwise.

8.1.3 Exploratory Hypothesis

8.1.3.1 Hypothesis- There is a possible association between the primary and secondary outcomes and immunological endpoints including serum IL-12 and IFN-γ levels, and NK cell cytolytic activity. Immunological measurements, as continuous variables, will be collected from blood at baseline and at specified times after the adenovirus injection.

Each variable will be evaluated for the proportional hazard assumption. Data will be categorized if the assumption is not met. Cox regression models will be used to study the association of baseline and post-treatment variables, respectively, with outcome. The analysis will begin testing the individual variable effect including testing for treatment by individual variable interaction, followed by multivariable analysis. The variable with individual effect at 0.05 level or variable interaction at 0.10 will remain in the final multivariable model with assessment of goodness-of-fit (e.g. ROC).

8.2 Sample Size Calculation/Power Justification for the Primary Endpoint

This is a MTD determination study. Therefore, the power calculation is not relevant. A 3 to 6 subject per dose cohort with a total of 15 to 30 subjects is standard for a phase 1 trial (72).

8.3 Statistical Analysis

Analysis for this phase 1 trial will be descriptive. We will report all toxicity results taking into account any baseline abnormalities.

9.0 DATA AND SAFETY MONITORING PLAN

A Data and Safety Monitoring Plan has been developed according to FDA guidelines (49592 Federal Register / Vol. 63, No. 179 / Wednesday, September 16, 1998 / Notices).

10.0 INCLUSION OF MINORITIES

Based on subject demographics of five phase 1/2 gene therapy trials conducted by the sponsor, the Targeted/Planned Enrollment for this clinical trial is shown in **Table 11**. Forty-five of the 102 (44%) subjects enrolled were minorities.

Table 11. Targeted/Planned Enrollment (assuming 15 subjects enrolled)

TARGETED/PLANNED ENROLLMENT: Number of Subjects			
ETHNIC CATEGORY	Gender		
	**FEMALES**	**MALES**	**TOTAL**
Hispanic or Latino	0	0	0
Not Hispanic or Latino	0	15	15
Ethic Categories: Total of All Subjects	0	15	15
RACIAL CATEGORIES			
American Indian/Alaskan Native	0	0	0
Asian	0	0	0
Native Hawaiian or Other Pacific Islander	0	0	0
Black or African American	0	7	7
White	0	8	8
Ethic Categories: Total of All Subjects	0	15	15

11.0 PROCESS OF INFORMED CONSENT

The Principal Investigator and Co-Principal Investigators will ensure that the study is conducted in full conformance with FDA standards for human research. An IRB-approved Informed Consent will be obtained for each subject. The form must be signed, witnessed, and dated. Copies of the signed document will be given to the subject and filed in the study Master File. All reports and subject samples will be identified only by a coded number to maintain subject confidentiality. All records will be kept confidential to the extent permitted by law.

12.0 LITERATURE CITED

1. American Cancer Society. Cancer Facts & Figures 2014. Atlanta: American Cancer Society; 2014.
2. Zelefsky M, Eastham J, Sartor O. Cancer of the prostate. In Cancer: Principles and Practice of Oncology, editors: Devita, Lawrence & Rosenberg, Lippincott Williams & Wilkins, 9th edition, 1220-1271 (2011).
3. Jones C, Hunt D, McGowan D *et al*. Radiotherapy and short-term androgen deprivation for localized prostate cancer. *N Engl J Med* 365: 107-118, 2011.
4. Marcus D, Canter D, Jani A et al. Salvage therapy for locally recurrent prostate cancer after irradiation. *Can J Urol* 19: 6534-6541, 2012.
5. Kimura M, Mouraviev V, Tsivian M *et al*. Current salvage methods for recurrent prostate cancer after failure of primary radiotherapy. *Brit J Urol Int*, 105: 191-201, 2009.
6. List of gene therapy clinical trials, OBA web site (http://www4.od.nih.gov/oba/rdna.htm).
7. Freeman S, Abboud C, Whartenby K, *et al*. The "bystander effect": tumor regression when a fraction of the tumor mass is genetically modified. *Cancer Res* 53: 5274-5283, 1993.
8. Bi W, Parysek L, Warnick R, *et al*. *In vitro* evidence that metabolic cooperation is responsible for the bystander effect observed with HSVtk retroviral gene therapy. *Hum Gene Ther* 4: 725-731, 1993.
9. Huber B, Austin E, Richards C, *et al*. Metabolism of 5-fluorocytosine to 5-fluorouracil in human colorectal tumor cells transduced with the cytosine deaminase gene: significant antitumor effects when only a small percentage of tumor cells express cytosine deaminase. *Proc Natl Acad Sci USA* 91: 8302-8306, 1994.
10. Moolten F, Wells J. Curability of tumors bearing herpes thymidine kinase genes transferred by retroviral vectors. *J Natl Cancer Inst* 82: 297-300, 1990.
11. Ezzeddine E, Martuza R, Platika D, *et al*. Selective killing of glioma cells in culture and in vivo by retrovirus transfer of the herpes simplex virus thymidine kinase gene. *The New Biol* 3: 608-614, 1991.
12. Culver K, Ram Z, Wallbridge S, *et al*. *In vivo* gene transfer with retroviral vector-producer cells for treatment of experimental brain tumors. *Science* 256: 1550-1552, 1992.
13. Takamiya Y, Short M, Moolten F, *et al*. An experimental model of retrovirus gene therapy for malignant brain tumors. *J Neurosurg* 79: 104-110, 1993.
14. Huber B, Austin E, Good S, *et al*. *In vivo* antitumor activity of 5-fluorocytosine on human colorectal carcinoma cells genetically modified to express cytosine deaminase. *Cancer Res* 53: 4619-4626, 1993.
15. Huber B, Austin E, Richards C, *et al*. Metabolism of 5-fluorocytosine to 5-fluorouracil in human colorectal tumor cells transduced with the cytosine deaminase gene: significant antitumor effects when only a small percentage of tumor cells express cytosine deaminase. *Proc Natl Acad Sci USA* 91: 8302-8306, 1994.
16. Mullen C, Coale M, Lowe R, *et al*. Tumors expressing the cytosine deaminase suicide gene can be eliminated in vivo with 5-fluorocytosine and induce protective immunity to wild type tumor. *Cancer Res* 54: 1503-1506, 1994.
17. Chen S-H, Shine H, Goodman J, *et al*. Gene therapy for brain tumors: regression of experimental gliomas by adenovirus-mediated gene transfer *in vivo*. *Proc Natl Acad Sci USA* 91: 3054-3057, 1994.
18. Richards C, Austin E, Huber B. Transcriptional regulatory sequences of carcinoembryonic antigen: identification and use with cytosine deaminase for tumor-specific gene therapy. *Hum Gene Ther* 6: 881-893, 1995.

19. Hirschowitz E, Ohwada A, Pascal W, *et al*. *In vivo* adenovirus-mediated gene transfer of the *Escherichia coli* cytosine deaminase gene to human colon carcinoma-derived tumors induces chemosensitivity to 5-fluorocytosine. *Hum Gene Ther* 6: 1055-1063, 1995.
20. Cool V, Pirotte B, Gerard C, *et al*. Curative potential of herpes simplex virus thymidine kinase gene transfer in rats with 9L gliosarcoma. *Hum Gene Ther* 7: 627-635, 1996.
21. Eastham J, Chen S-H, Sehgal I, *et al*. Prostate cancer gene therapy: herpes simplex virus thymidine kinase gene transduction followed by ganciclovir in mouse and human prostate cancer models. *Hum Gene Ther* 7: 515-523, 1996.
22. Dong Y, Wen P, Manome Y, *et al*. *In vivo* replication-deficient adenovirus vector-mediated transduction of the cytosine deaminase gene sensitizes glioma cells to 5-fluorocytosine. *Hum Gene Ther* 7: 713-720, 1996.
23. Ohwada A, Hirschowitz E, Crystal R. Regional delivery of an adenovirus vector containing the *E. Coli*. cytosine deaminase gene to provide local activation of 5-fluorocytosine to suppress the growth of colon carcinoma metastatic to liver. *Hum Gene Ther* 7: 1567-1566, 1996.
24. Hall S, Mutchmik S, Chen S-H, *et al*. Adenovirus-mediated herpes simplex virus thymidine kinase gene and ganciclovir therapy leads to systemic activity against spontaneous and induced metastasis in an orthotopic mouse model of prostate cancer. *Int J Cancer* 70: 183-187, 1997.
25. Hall S, Mutchnik S, Yang G, *et al*. Cooperative therapeutic effects of androgen ablation and adenovirus-mediated herpes simplex virus thymidine kinase gene and ganciclovir therapy in experimental prostate cancer. *Can Gene Ther* 6: 54-63, 1998.
26. Kim J-H, Kim S-H, Brown S, *et al*. Selective enhancement by an antiviral agent of the radiation-induced cell killing of human glioma cells transduced with HSV-tk gene. *Cancer Res* 54, 6053-6056, 1994.
27. Kim J-H, Kim S-H, Kolozsvary A, *et al*. Selective enhancement of radiation response of herpes simplex virus thymidine kinase transduced 9L gliosarcoma cells *in vitro* and *in vivo* by antiviral agents. *Int J Radiat Oncol Biol Phys* 33: 861-868, 1995.
28. Khil M, Kim J-H, Mullen C, *et al*. Radiosensitization by 5-fluorocytosine of human colorectal carcinoma cells in culture transduced with cytosine deaminase gene. *Clin Cancer Res* 2: 53-57, 1996.
29. Rogulski K, Kim J-H, Kim S-H, *et al*. Glioma cells transduced with an *E. coli* CD/HSV-1 TK fusion gene exhibit enhanced metabolic suicide and radiosensitivity. *Hum Gene Ther* 8: 73-85, 1997.
30. Rogulski K, Zhang K, Kolozsvary A, *et al*. Pronounced anti-tumor effects and tumor radiosensitization of double suicide gene therapy. *Clin Cancer Res* 3: 2081-2088, 1997.
31. Kim J-H, Kolozsvary A, Rogulski K, *et al*. Double suicide fusion gene: a selective radiosensitization of 9L glioma in rat brain. *Can J Scient Am* 4: 364-369, 1998.
32. Xie Y, Gilbert J, Kim J-H, *et al*. Efficacy of adenovirus-mediated CD/5-FC and HSV-1 TK/GCV suicide gene therapies concomitant with p53 gene therapy. *Clin Cancer Res* 5: 4224-4232, 1999.
33. Freytag S, Rogulski K, Paielli D, *et al*. A novel three-pronged approach to selectively kill cancer cells: concomitant viral, double suicide gene, and radiotherapy. *Hum Gene Ther* 9, 1323-1333, 1998.
34. Rogulski K, Wing M, Paielli D, *et al*. Double suicide gene therapy augments the therapeutic efficacy of a replication-competent lytic adenovirus through enhanced cytotoxicity and radiosensitization. *Hum Gene Ther* 11: 67-76, 2000.
35. Freytag S, Paielli D, Wing M, *et al*. Efficacy and toxicity of replication-competent adenovirus-mediated double suicide gene therapy in combination with radiation therapy in an orthotopic mouse prostate cancer model. *Int J Radiat Oncol Biol Phys* 54: 873-886, 2002.

36. Freytag S, Khil M, Stricker H, *et al*. Phase I study of replication-competent adenovirus-mediated double suicide gene therapy for the treatment of locally recurrent prostate cancer. *Cancer Res* 62: 4968-4976, 2002.

37. Freytag S, Stricker H, Pegg J, *et al*. Phase I study of replication-competent adenovirus-mediated double suicide gene therapy in combination with conventional dose three-dimensional conformal radiation therapy for the treatment of newly-diagnosed, intermediate-to high-risk prostate cancer. *Cancer Res* 63: 7497-7506, 2003.

38. Freytag S, Stricker H, Peabody J, *et al*. Five-year follow-up of trial of replication-competent adenovirus-mediated suicide gene therapy for treatment of prostate cancer. *Mol Ther* 15: 636-642, 2007.

39. Freytag S, Movsas B, Aref I, *et al*. Phase I trial of replication-competent adenovirus-mediated suicide gene therapy with IMRT for prostate cancer. *Mol Ther* 15: 1016-1023, 2007.

40. Freytag S, Stricker H, Movsas B, *et al*. Prostate cancer gene therapy clinical trials. *Mol Ther* 15: 1042-1052, 2007.

41. Freytag S, Stricker H, Movsas B, *et al*. Gene therapy for prostate cancer, in *Gene-based therapies for cancer*, Roth JA, ed., Springer, New York (2010).

42. Barton K, Stricker H, Brown S, *et al*. Phase I study of noninvasive imaging of adenovirus-mediated gene expression in the human prostate. *Mol Ther* 16: 1761-1769, 2008.

43. Barton K, Stricker H, Elshaikh M, *et al*. Feasibility of adenovirus-mediated hNIS gene transfer and ^{131}I radioiodine therapy as a definitive treatment for localized prostate cancer. *Mol Ther* 19: 1353-1359, 2011.

44. Freytag S, Stricker H, Lu M, Elshaikh M, Aref I, Pradhan D, Levin K, Kim JH, Peabody J, Siddiqui F, Barton K, Pegg J, Zhang Y, Cheng J, Oja-Tebbe N, Bourgeois R, Gupta N, Lane Z, Rodriguez R, DeWeese T, Movsas B. Prospective randomized phase 2 trial of intensity modulated radiation therapy with or without oncolytic adenovirus-mediated cytotoxic gene therapy in intermediate-risk prostate cancer. *Int J Rad Oncol Biol Phys* 89: 268-276, 2014.

45. Barton K, Paielli D, Zhang Y, *et al*. Second-generation replication-competent oncolytic adenovirus armed with improved suicide genes and the ADP gene demonstrate greater efficacy without increased toxicity. *Mol Ther* 13: 347-356, 2006.

46. Kievit E, Bershad E, Ng E, *et al*. Superiority of yeast over bacterial cytosine deaminase for enzyme/prodrug gene therapy in colon cancer xenografts. *Cancer Res* 59: 1417-1421, 1999.

47. Black M, Kokoris M, Sabo, P. Herpes simplex virus-1 thymidine kinase mutants created by semi-random sequence mutagenesis improve prodrug-mediated tumor cell killing. *Cancer Res* 61: 3022-3026, 2001.

48. Tollefson A, Scaria A, Hermiston T, *et al*. The adenovirus death protein (E3-11.6 K) is required at very late stages of infection for efficient cell lysis and release of adenovirus from infected cells. *J Virol* 70: 2296-2306, 1996.

49. Tollefson A, Ryerse J, Scaria A, *et al*. The E3-11.6 kDa adenovirus death protein (ADP) is required for efficient cell death: characterization of cells infected with adp mutants. *Virology* 220: 152-162, 1996.

50. Barton K, Tyson D, Stricker H, et al. GENIS: gene expression of sodium iodide symporter for non-invasive imaging of gene therapy vectors and quantification of gene expression *in vivo*. *Mol Ther* 8: 508-518, 2003.

51. Freytag S, Barton K, Zhang Y, *et al*. Replication-competent adenovirus-mediated suicide gene therapy with radiation in a preclinical model of pancreatic cancer. *Mol Ther* 15: 1600-1606, 2007.

52. Sandler H, Dunn R, McLaughlin P, *et al*. Overall survival after prostate-specific-antigen-detected recurrence following conformal radiation therapy. *Int J Radiat Oncol Biol Phys* 48:

629-633, 2000.

53. Kuban D, el-Mahdi A, Schellhammer P. The significance of post-irradiation prostate biopsy with long-term follow-up. *Int J Radiat Oncol Biol Phys* 24: 409-414, 1992.
54. Zelefsky M, Reuter V, Fuks Z, *et al*. Influence of local tumor control on distant metastases and cancer related mortality after external beam radiotherapy for prostate cancer. *J Urol* 179: 1368-1373, 2008
55. Crook J, Malone S, Perry G, *et al*. Twenty-four-month postradiation prostate biopsies are strongly predictive of 7-year disease-free survival: results from a Canadian randomized trial. *Cancer* 115: 673-679, 2009.
56. Cannistra S. Phase II trials in *Journal of Clinical Oncology*. *J Clin Oncol* 27: 3073-3076, 2009.
57. Freytag S, Barton K, Zhang Y. Efficacy of oncolytic adenovirus expressing suicide genes and interleukin 12 in preclinical model of prostate cancer. *Gene Therapy* 20: 1131-1139, 2013.
58. Trinchieri G. Interleukin-12 and the regulation of innate resistance and adaptive immunity. *Nat Rev Immunol* 3: 133-146, 2003.
59. Del Vecchio M, Bajetta E, Canova S, *et al*. Interleukin 12: Biological properties and clinical application. *Clin Cancer Res* 13: 4677-4685, 2007.
60. Colombo M, Trinchieri G. Interleukin-12 in antitumor immunity and immunotherapy. *Cytokine Growth Factor Rev* 13: 155-168, 2002.
61. Nasu Y, Bangma C, Hull G, *et al*. Adenovirus-mediated interleukin-12 gene therapy for prostate cancer: suppression of orthotopic tumor growth and pre-established lung metastases in an orthotopic model. *Gene Ther* 6: 338-349, 1999.
62. Atkins M, Robertson M, Gordon M, *et al*. Phase I evaluation of intravenous recombinant human interleukin 12 in patients with advanced malignancies. *Clin Cancer Res* 3: 409-417, 1997.
63. Leonard J, Sherman M, Fisher G, et al. Effects of single-dose interleukin-12 exposure on interleukin-12 associated toxicity and interferon-γ production. *Blood* 90: 2541-2548, 1997.
64. Wei J, Dunn R, Litwin M, *et al*. Development and validation of the expanded prostate cancer index composite (EPIC) for comprehensive assessment of health-related quality of life in men with prostate cancer. *Urology* 56: 899-905, 2000.
65. van Andel G, Kurth K. The impact of androgen deprivation therapy on health related quality of life in asymptomatic men with lymph node positive prostate cancer. *Eur Urol* 44: 209-214, 2003.
66. Rabin R, de Charro F. EQ-5D: a measure of health status from the EuroQol Group. *Ann Med* 33: 337-343, 2001.
67. *NIH Guidelines for Research Involving Recombinant or Synthetic Nucleic Acid Molecules.* http://www4.od.nih.gov/oba/rac/guidelines/guidelines.html.
68. http://www.hrc.govt.nz/sites/default/files/CTCAE%20manual%20-%20DMCC.pdf.
69. http://www.fda.gov/safety/medwatch/howtoreport/ucm053087.htm
70. http://ctep.cancer.gov/protocolDevelopment/electronic_applications/docs/aeguidelines.pdf
71. Roach M, Hanks G, Thames H, *et al*. Defining biochemical failure following radiotherapy with or without hormone therapy in men with clinically localized prostate cancer: recommendations of the RTOG-ASTRO Phoenix Consensus Conference. *Int J Radiat Oncol Biol Phys* 65: 965-974, 2006.
72. Piantadosi, S. Clinical Trials, A Methodologic Perspective. New York: John Wiley & Sons, Inc., 1997.
