# Supplementary material for: A phase I clinical trial of oncolytic adenovirus mediated suicide and interleukin-12 gene therapy in patients with recurrent localized prostate adenocarcinoma
Source: PLoS One. 2023 Sep 15;18(9):e0291315. doi: 10.1371/journal.pone.0291315 (PMC10503775; doi:10.1371/journal.pone.0291315)
Supplement: S2 File — (PDF) [file pone.0291315.s004.pdf]

REQUEST FOR PLANNED CHANGE(S)

Changes **may not** be implemented until IRB written approval is received.
Investigators are responsible for utilizing the most current versions of IRB forms and the IRB has the authority to refuse out of date forms.

This form can now be submitted electronically! See general directions at end of form!

Principal Investigator (PI): **Hans Stricker, MD/Svend Freytag, PhD**

Department (select from the drop downs): **Radiation Oncology** Division:

Entire Project Title (no acronyms): **Phase 1 Trial of Oncolytic Adenovirus-Mediated Cytotoxic and Interleukin 12 Gene Therapy for locally Recurrent Prostate Cancer After Definitive Radiotherapy**

IRB #.: **9829** Current IRB Approval Period: - Location to send correspondence (required): **OFP** 5D-39
Contact Person: **Janice Freytag** Contact phone #: **74-8326** Contact e-mail: **jfreyta1@hfhs.org**

Reason for IRB review (check all that apply)**:**

1. ☐ Protocol Amendment:
2. ☐ Investigator Brochure Update/Package insert:
3. ☒ Response to IRB Concerns from:
   - ☒ Initial Submission
   - ☐ Previous Planned Change Form
   - ☐ Adverse Event Form
   - ☐ Continuation/final report un-approved
   - ☐ Other:
4. ☒ Revised Consent Form:**6.2**
5. ☐ Advertisement
6. ☐ PI Change (will require revised consent form)
7. ☐ Title Change (will require revised consent form)
8. ☐ Database Declaration
9. ☐ DSMB Reports (attach report)
10. ☐ Protocol Deviation:
11. ☐ Request to Re-open a closed study
12. ☐ Study closed to accrual:
13. ☐ Other:

THE REST OF THIS PAGE IS FOR IRB USE ONLY

Type of IRB Review:
☒ Expedited ☐ Full Board (reviewed at a Full Board Meeting on:)

Risk to subjects increased: ☐ Yes ☒ No
Consent form satisfactorily revised: ☒Yes ☐ No ☐ N/A

If yes, does this require re-consenting ***currently active*** *subjects in treatment phase:* ☐ Yes ☐ No
If yes, does this require re-consenting ***all*** *subjects:* ☐ Yes ☐ No

Result of IRB Review:
☒ Approve
☐ Withheld pending response (☐ Member review ☐ Administrative review)
☐ Approval denied (requires full board review)
☐ IRB notified: Approval not required

APPROVAL STAMP
APPROVED<br>Aug 19, 2015<br>INSTITUTIONAL REVIEW BOARD

The HFHS IRB has read & reviewed this protocol & finds that this research is appropriate in design and meets the requirements of the Federal Guidelines, 45 CFR Part 46 and 21 CFR Part 50. The signature below denotes IRB approval of this study.

Timothy Roehrs PhD
Chairperson or designee - Henry Ford Health System IRB

Date: **8-19-15**

Comments from Chairperson or designee:
Action required:

<u>COMPLETE THE FOLLOWING QUESTIONS AS DIRECTED</u>
<u>(if your item number is not noted, you only need to complete numbers 1-5 on this page)</u>

1. Provide a description/explanation of this submission. ("See attached" format is not acceptable) **The purpose of this planned change form is to repond to the requests of the IRB:**

   1. Provide the IRB a copy of the approval letter from the IRDBC

   2. Provide the IRB a revised consent form outlining the risks of the study stratified by "likely", "less likely" and "rare".

2. Does this change increase the risk significantly? ☒ **No** ☐ **Yes** (consent changes may be required)

3. How many HFHS subjects are currently enrolled? **0** Is the study still enrolling subjects at HFHS?
   - ☐ **No**
     - Are there subjects still in the active treatment phase of the study? ☐ No ☐ Yes
     - Are there subjects still in the long term follow-up phase of the study? ☐ No ☐ Yes
   - ☐ Yes

4. Does the benefit still outweigh the risk for this study? ☐ **No** ☒ **Yes**

5. Does the new information require a revised informed consent?
   - ☐ **No**
     - o If no, why not?
     - o Was consent for this study waived originally? ☐ **No** ☐ **Yes**
   - ☒ **Yes** - submit 2 copies of the consent form with one yellow highlighted to show the changes

6. If you checked numbers **<u>1 - 2</u>** on page one, answer the following. If not, proceed to the next question.
   - Is the study sponsored? ☒ **No** ☐**Yes** Name of Sponsor (if applicable):

     If yes, check the appropriate box and provide the information requested:
     - ☐ A copy of the sponsor's amendment, if sponsor initiated the change.
     - ☐ A copy of your notice to the sponsor, if you initiated the change.
     - ☐ Other:

7. If you checked number **<u>3</u>** on page one, attach the copy of the IRB letter you received, your response, and the revised consent form (attach 2 copies of the revised consent, all consent changes must be marked with a yellow highlighting on one copy) if applicable. If not, proceed to the next question.

8. If you checked number **<u>5</u>** on page one, complete the following. If not, proceed to the next question.
   - Advertisements must be submitted for review before being published. Please include the reasons for the advertisement, and where it will be placed.
   - If you are submitting an advertisement for television or radio, please attach a copy of the script along with a video or audiotape if available.
   - If you are submitting an advertisement for the HFHS external Internet, please complete the following and attach the sponsor's approval of the text of the advertisement as a supporting document.
     - Date you want trial on web site:
     - Date you want trial off web site:
     - Choose your clinical area: If 'other', please specify:
     - In lay terms, please indicate the eligibility criteria for subjects:
     - How do you want patients to contact you to get into the trial (ie. name & phone #)?
     - In lay terms, describe the purpose of the study (you can use the sentence from your consent form:
     - If the study is sponsored, please indicate the sponsor name:
     - If you know the grant number, please provide:

9. If you checked number **<u>6</u>** on page one, attach letter from the new PI indicating they will assume the responsibility for the study and the signatures of both the current & new investigator. You must also change the PI in the consent form (if applicable), and submit. If not, proceed to the next question.

10. If you checked number **<u>7</u>** on page one, type the old & new title in the title section of page 1, & highlight the new title, you must also change the title on the consent form (if applicable), & submit it for review. If not, proceed to the next question.

11. If you checked number **10** on page one, submit only significant deviations as deemed by the investigator. If not, proceed to the next question.

12. If you checked number **11** on page one, please indicate the reason below. If not, proceed to the next question.
    - ☐ Audit Site Visit
    - ☐ Query for data clarification/data existing at the time of study closure
    - ☐ Query for new data related to events occurring since study closure
    - ☐ To Notify Subjects of their Randomization and the Study Results (attach the letter)
    - ☐ Protocol lapsed and was administratively closed (provide reason for lapse, any study-related unanticipated problems that occurred since the study closed, and plan to prevent a lapse from occurring in the future).

General Information for completing this form

1. This form must be typed with all blank shaded spaces completed. You can tab through the form, entering your information in the spaces provided. Please use attachment pages when requested, providing all the required and necessary information.
2. You may submit this request either paper or electronically (but not a combination of both). If you submit electronically, all supporting documents must also be submitted electronically and be saved in word or adobe.

To submit by paper	To submit Electronically
Submit this original signed form and any attachments, along with *1 extra copy* (of this form and any attachments) to the Research Administration (IRB) Office (CFP Basement room 46). Please copy the packet either all front side only or all both sides (not a combination).	E-mail this completed form **and** supporting documents to: research_admin@hfhs.org You must follow these directions:<br>• The e-mail subject line must be in the following format (with underscore):<br>a. Indicate 'IRB PCF'<br>b. PI last name<br>c. HFHS IRB #<br>d. ***Example:*** 'IRB PCF_Jones_123'<br>• Attach the *Planned Change Form* saved with a title of: PCF and PI last name and IRB number (i.e. 'PCF_jones_123'). Note use an underscore between the items.<br>• *If applicable:* attach supporting documents saved with PCF, PI last name, IRB number and 'supportingdoc' as the title. If there is more than one supporting document, differentiate them by number (supportingdoc_1, supportingdoc *2,* ICF_3, etc.). *example:* PCF_Jones_123_supportingdoc_1<br><br>Your forms will be returned to you by e-mail. You may print them for your records as you will **not** be receiving copies through interdepartmental mail. As always, should you need a copy, the IRB maintains paper files for every study.

All IRB forms are available through the Research Administration website (http://henry.hfhs.org/body.cfm?id=166).
